# Silymarin and serotonin, individually and synergistically, enhance fenugreek resistance to salt stress by modulating pathways involved in chlorophyll biosynthesis, defense system, hormonal regulation, ion redox balance, and diosgenin production

**DOI:** 10.1186/s12870-026-08765-7

**Published:** 2026-04-17

**Authors:** Sajedeh Roshandel, Amin Ebrahimi, Shahrokh Gharanjik

**Affiliations:** 1 Agronomy and Plant Breeding Department, Faculty of Agriculture, Shahrood University of Technology, Semnan, Iran; 2 Department of Plant Breeding and Biotechnology, Faculty of Agricultural Engineering, Shahrood University of Technology, Shahrood, Iran

**Keywords:** Silymarin, Serotonin, Salinity stress, Exogenous biostimulants, Fenugreek, Diosgenin content

## Abstract

**Background:**

Salinity stress, as one of the most significant and widespread abiotic stresses, affects a large portion of the world’s arable lands. Developing salt-tolerant plants through classical breeding, biotechnology, and molecular methods is time-consuming and expensive. Considerable research attention has been directed toward the application of exogenous biostimulants as potential strategies to mitigate the adverse effects of salinity. The current research sought to investigate the impacts of varying silymarin (0, 250, and 500 µM) and serotonin (0, 50, and 100 µM) concentrations on the physiological, biochemical, and molecular responses of fenugreek under salt stress conditions (200 mM).

**Results:**

Salinity-induced oxidative stress adversely affected physiological parameters, including photosynthetic pigments, K^+^ concentration, relative water content, and auxin levels. Conversely, it triggered an upregulation in abscisic acid and nitric oxide levels, along with increased activity of both enzymatic and non-enzymatic antioxidants, accumulation of compatible osmolytes, and elevated cellular Na^+^ content. The enhancement of fenugreek tolerance to salt stress by these two compounds was associated with their ability to modulate key physiological and biochemical pathways. These findings are likely attributable to the reduction in Na^+^ content, as well as decreased hydrogen peroxide concentrations, which contribute to maintaining membrane integrity and cellular turgor, and reducing lipid peroxidation and ELI. Moreover, these included the regulation of photosynthetic pigment biosynthesis, abscisic acid and auxin levels, and the signaling molecules, including nitric oxide and hydrogen peroxide. Furthermore, the utilization of these two elicitors significantly enhanced the expression of genes associated with the diosgenin biosynthesis pathway, resulting in increased diosgenin content.

**Conclusions:**

Our results lay the groundwork for further investigation into the molecular mechanisms of plant defense mediated by these compounds, providing valuable perspectives on novel approaches to crop cultivation that leverage these two plant growth regulators.

**Supplementary Information:**

The online version contains supplementary material available at 10.1186/s12870-026-08765-7.

## Background

Soil salinity is widely recognized as a critical threat to crop growth and yield. Decreases in plant growth and yield associated with soil salinity may arise from modifications in a range of physiological and biochemical characteristics. These changes may include decreased leaf chlorophyll and carotenoid content, diminished photosynthetic capacity, and shifts in energy dynamics related to ion removal mechanisms, osmotic regulation, and nutrient imbalances [1]. As the global population continues to grow and arable land becomes increasingly limited, addressing the issue of soil salinity is imperative. Consequently, the development of salt-tolerant plants is of paramount importance and necessitates increased research efforts in this area [2]. Recent research indicates that neurotransmitters and biostimulants play a crucial role in key physiological processes in plants. These compounds have been demonstrated to regulate ion transport, mediate stomatal behavior, modulate reactive oxygen species (ROS) accumulation, and influence gene expression [3–5]. Furthermore, they play a crucial role in enhancing antioxidant defense systems and activating stress-responsive signaling pathways, thereby contributing to plant tolerance to abiotic stress [6]. The application of exogenous biostimulants represents a cost-effective strategy for inducing salinity tolerance genes and enhancing plant adaptation to saline environments, making it an appealing approach for plant breeders [6–9].

Serotonin, also referred to as 5-hydroxytryptamine, is a well-characterized metabolite synthesized from tryptophan [10]. The involvement of serotonin in plant responses to diverse stresses is well-documented [11–13]. Serotonin exhibits significant antioxidant properties, effectively mitigating the damaging effects of ROS and thereby providing protection against diverse environmental stresses. Additionally, serotonin influences the expression of various stress-related metabolic pathways, thereby modulating plant hormone signaling networks [14].

Silymarin is a flavonolignan that has recently been identified as a hepatoprotective agent. While silymarin is recognized for its diverse pharmacological and biological activities in both animals and humans [15], there is a paucity of scientific literature regarding its potential as a growth promoter in major crops and its overall effects on plant physiology. Recently, silymarin has been utilized either independently or as an additive to enhance plant biostimulants, aiming to alleviate the detrimental impacts of environmental stress [7, 16]. As a secondary metabolite and a bioactive antioxidant, silymarin serves a crucial role in plant resilience. Its exogenous supplementation has been shown to significantly improve the performance of stressed plants by bolstering their defense mechanisms against ROS, thereby offering substantial protection against oxidative damage [17]. Previous research has demonstrated that seed priming and foliar application of silymarin effectively mitigate the toxic effects of cadmium stress in *Phaseolus vulgaris* [18]. Nonetheless, more extensive research is necessary to clarify the mechanisms through which silymarin improves plant stress resilience [18].

Fenugreek (*Trigonella foenum-graecum* L.) is recognized as one of the oldest cultivated medicinal plants in documented history. This plant is rich in numerous bioactive compounds, including flavonoids, steroids, alkaloids (such as trigonelline, gentianine, and choline), saponins (notably thiogeno diosgenite), volatile oils, and lysine-rich proteins [19]. The seeds of fenugreek are particularly rich in essential vitamins, including A, B_1_, B_2_, C, niacin, nicotinic acid, pyridoxine, calcium pantothenate, biotin, ascorbic acid, and diosgenin. Diosgenin is a steroidal sapogenin classified within the triterpene group and is a significant metabolite extracted from various plant species, including *Dioscorea* spp., *Smilax china*, and *Trigonella foenum-graecum*. Diosgenin has been utilized in the treatment of multiple disorders, including diabetes, hyperlipidemia, rheumatoid arthritis, leukemia, and cancer. Additionally, diosgenin is recognized for its potential in the production of sex hormones, contraceptives, and other steroid-derived compounds [20, 21].

Given the escalating prevalence of salt stress globally, coupled with the imperative to develop resistant cultivars, there is a pressing need for innovative, efficient, cost-effective, and environmentally sustainable methods to mitigate stress-induced conditions [22]. Although previous studies have reported that silymarin and serotonin can aid plants in coping with various stresses, their synergistic effects on growth and productivity under salt stress have not been thoroughly investigated to date. Furthermore, elucidating the mechanisms underlying plant responses to silymarin and serotonin is crucial for developing strategies aimed at enhancing crop resilience [23, 24]. The current research sought to clarify the operational mechanisms of serotonin and silymarin in fenugreek by assessing physiological, biochemical, and molecular characteristics that confer resistance to salt stress. Additionally, the study evaluated the expression of specific genes implicated in the diosgenin biosynthesis pathway in fenugreek treated with serotonin and silymarin under saline conditions. Furthermore, an additional aim of this investigation was to probe the interactions among various plant hormones, specifically auxin and abscisic acid (ABA), alongside key signaling molecules such as nitric oxide (NO) and hydrogen peroxide (H_2_O_2_), in conjunction with serotonin and silymarin.

## Results

### Analysis of variance

The results of the variance analysis table for the traits investigated in this study indicated that the main effects of all three treatments—salinity stress, varying concentrations of serotonin, and silymarin—were statistically significant at the 1% probability level for all traits examined. Furthermore, the dual interaction effects among the studied traits were significant for the majority of the evaluated traits. Specifically, the dual interaction between different concentrations of serotonin and silymarin was significant for all traits studied, except for chlorophyll content, carotenoid content, relative water content (RWC), malondialdehyde content (MDA), antioxidant enzymes (such as ascorbate peroxidase (APX) and guaiacol peroxidase (GPX), ABA content, NO content, and the squalene synthase reductase (*SSR*), sterol methyltransferase (*SMT*), and 26-o-Beta glucosidase (*BGL*) genes. Additionally, the dual interaction effect of salinity stress and varying concentrations of silymarin was significant for all traits assessed in this study (except for *SSR*, and *SMT* genes). Similarly, the dual interaction between salinity stress and different concentrations of serotonin was significant for all evaluated traits, except chlorophyll content, potassium (K^+^), ABA content, squalene epoxidase (*SEP*), *SMT*, and *SSR* genes. The triple interaction effect of salinity stress, varying concentrations of silymarin, and serotonin was significant for several traits, including RWC, electrolyte leakage index (ELI), total soluble protein content, antioxidant enzymes (such as superoxide dismutase (SOD)), and polyphenol oxidase (PPO)), total phenol and flavonoid content, sodium content (Na^+^), H_2_O_2_, squalene synthase (*SQS)*, cycloartenol synthase (*CAS*), and *BGL* genes as reported in Supplementary Table 1.

### Photosynthetic pigments and relative water content

Analysis of photosynthetic pigments in response to varying silymarin concentrations under saline conditions revealed a pronounced stress-induced degradation of these compounds. In plants subjected to salinity without silymarin application, a marked and significant decline in pigment content was observed relative to the control group. Specifically, levels of chlorophyll a, chlorophyll b, total chlorophyll, and carotenoids were diminished by 47%, 46%, 45%, and 42%, respectively. Conversely, The application of 500 µM silymarin in fenugreek plants under salinity stress significantly enhanced chlorophyll a, chlorophyll b, total chlorophyll, and carotenoid content by 47%, 70%, 53%, and 78%, respectively, compared to plants under salinity stress without silymarin treatment (Table 1).

**Table 1:** Comparison of the mean dual interaction effects of varying salinity levels and silymarin concentrations on the photosynthetic pigments content

Salinity (mM)	Silymarin concentrations (µM)	chlorophyll a (mg g^− 1^ FW)	chlorophyll b (mg g^− 1^ FW)	Total chlorophyll (mg g^− 1^ FW)	Carotenoid (mg g^− 1^ FW)
0	0	19.77 ± 0.84 c	5.97 ± 0.27 c	25.75 ± 1.11 c	5.32 ± 0.22 d
0	250	21.22 ± 0.61 b	6.80 ± 0.32 c	28.02 ± 0.92 b	5.87 ± 0.23 b
0	500	22.38 ± 0.69 a	7.25 ± 0.30 a	29.63 ± 0.97 a	7.07 ± 0.28 a
200	0	10.55 ± 0.98 f	3.52 ± 0.30 e	14.07 ± 1.28 f	3.11 ± 0.28 f
200	250	13.11 ± 0.72 e	4.75 ± 0.35 d	17.86 ± 1.06 e	4.26 ± 0.29 e
200	500	15.44 ± 0.76 d	6.08 ± 0.30 c	21.53 ± 1.06 d	5.55 ± 0.30 c

Mean comparisons were performed employing Duncan’s multiple range test for statistical analysis. The findings are reported as mean values ± standard deviation, derived from three replicate measurements. Columns sharing identical alphabetical superscripts do not differ significantly at *p* ≤ 0.01

**Fig. 1:**
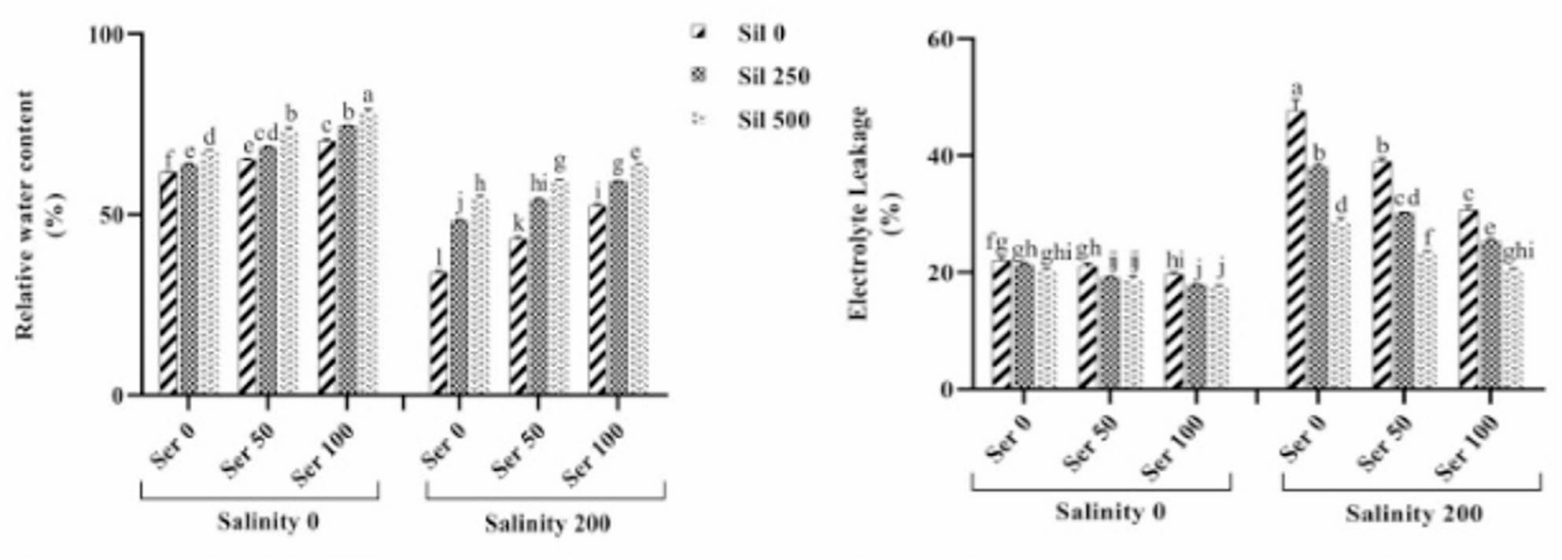
Comparison of the mean triple interaction effects of varying salinity levels (0 and 200 mM), silymarin (0, 250, and 500 µM) (Sil), and serotonin concentrations (0, 50, and 100 µM) (Ser) on the relative water content and electrolyte leakage index. Comparisons of means were performed using Duncan’s multiple range test. Bars sharing identical letters signify no statistically significant differences between treatments

An assessment of the triple interaction effects of salinity stress, serotonin, and silymarin varying concentrations on the RWC demonstrated that exposure to saline conditions in the absence of both serotonin and silymarin resulted in a significant 45% decrease in this parameter compared to non-stressed conditions. Notably, the combined application of 100 µM serotonin and 500 µM silymarin to plants cultivated under salinity stress enhanced the RWC by 85% relative to stressed plants that were not treated with these elicitors (Fig. 1).

### Malondialdehyde content and electrolyte leakage index

**Fig. 2:**
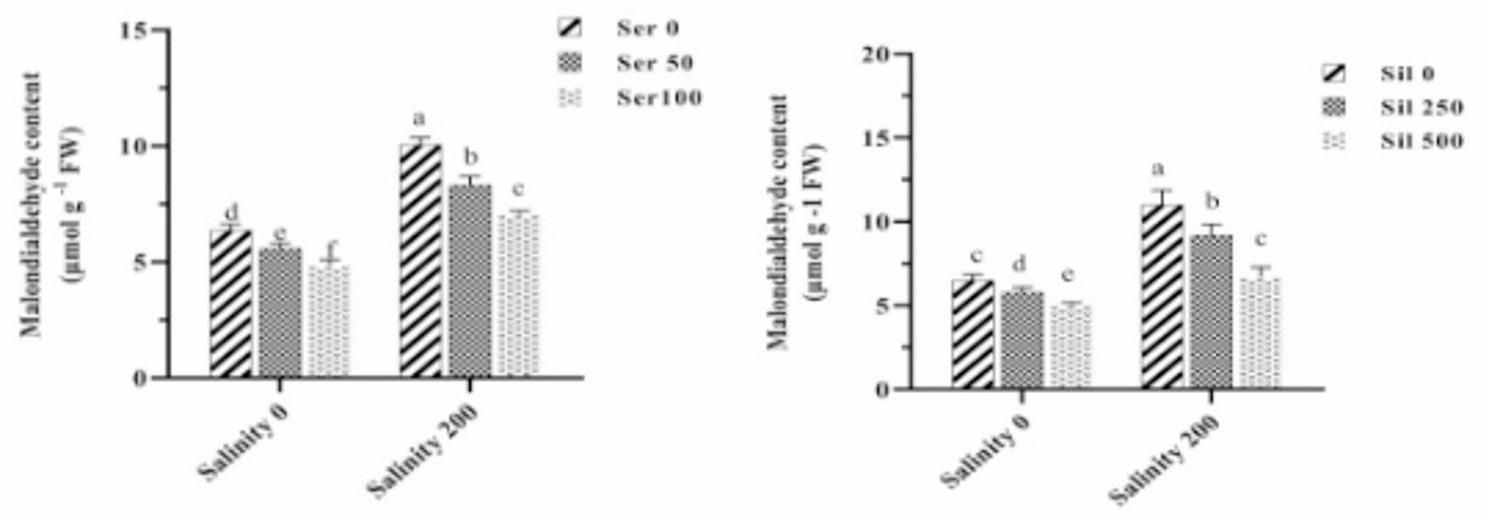
Comparison of the mean dual interaction effects of varying salinity levels (0 and 200 mM) at silymarin (Sil) (0, 250, and 500 μM), and salinity at serotonin (Ser) (0, 50, and 100 μM) on the malondialdehyde content. Comparisons of means were performed using Duncan’s multiple range test. Bars sharing identical letters signify no statistically significant differences between treatments

The mean comparison of the triple interaction effects of salinity stress, varying levels of silymarin, and serotonin revealed that, under salinity stress without the application of these two elicitors, the ELI significantly increased by 116% compared to normal conditions. Concurrent treatment of plants under salinity stress with 100 µM serotonin and 500 µM silymarin resulted in a 60% reduction in the ELI compared to plants under salinity stress without these compounds (Fig. 1). The mean comparison of the salinity stress effects at silymarin different levels and salinity stress at serotonin various concentrations indicated that exposure to saline conditions in the absence of either compound led to a significant elevation in MDA content relative to group control. However, application of 100 µM serotonin or 500 µM silymarin to plants cultivated under salinity stress reduced MDA levels by 38% and 31%, respectively, compared to stressed plants that received neither treatment (Fig. 2).

### Soluble protein content and antioxidant enzyme activity

Comparison of the mean effects of the three-way salinity stress interaction across serotonin different levels and silymarin on the soluble protein content revealed that salinity stress without application of these two elicitors significantly increased soluble protein content by 89% in contrast to normal conditions. Additionally, the simultaneous application of 100 µM serotonin and 500 µM silymarin under salinity stress resulted in an 87% increase in this trait compared to the absence of these elicitors under salinity stress (Table 2).

**Table 2:** Comparison of the mean effects of the triple interaction between salinity levels, serotonin, and silymarin concentrations on the soluble protein content, superoxide dismutase and polyphenol oxidase activities

Salinity (mM)	Silymarin concentrations (µM)	Serotonin concentrations (µM)	Soluble protein (mg ml^− 1^ protein)	Superoxide dismutase (µmol min^− 1^ mg^− 1^ protein)	Polyphenol oxidase (µmol min^− 1^ mg^− 1^ protein)
0	0	0	123.66 ± 2.02 n	0.03633 ± 0.00033 j	0.02900 ± 0.00057 k
0	0	50	135.00 ± 2.89 lm	0.04533 ± 0.00120 i	0.03900 ± 0.00100 ij
0	0	100	148.66 ± 1.32 jk	0.05766 ± 0.00233 h	0.04916 ± 0.00098 h
0	250	0	129.33 ± 2.33 mn	0.04700 ± 0.00173 i	0.03300 ± 0.00115 jk
0	250	50	143.33 ± 3.33 kl	0.05633 ± 0.00185 h	0.04466 ± 0.00120 hi
0	250	100	159.33 ± 2.33 i	0.06833 ± 0.00166 g	0.05933 ± 0.00066 g
0	500	0	140.66 ± 2.33 kl	0.05680 ± 0.00041 h	0.03773 ± 0.00115 j
0	500	50	157.33 ± 1.45 ij	0.06766 ± 0.00145 g	0.04820 ± 0.00091 h
0	500	100	171.33 ± 1.32 h	0.07866 ± 0.00088 f	0.06200 ± 0.00152 fg
200	0	0	233.33 ± 3.33 g	0.07600 ± 0.00115 f	0.06700 ± 0.00152 f
200	0	50	296.66 ± 3.33 f	0.09366 ± 0.00033 e	0.08400 ± 0.00173 e
200	0	100	376.66 ± 6.67 cd	0.12800 ± 0.00200 d	0.10733 ± 0.00266 d
200	250	0	307.33 ± 2.66 e	0.09700 ± 0.00153 e	0.08166 ± 0.00088 e
200	250	50	369.66 ± 5.49 d	0.13100 ± 0.00100 d	0.11577 ± 0.00333 c
200	250	100	400.33 ± 0.32 b	0.16733 ± 0.00145 b	0.15433 ± 0.00285 b
200	500	0	379.66 ± 5.49 c	0.12900 ± 0.00057 d	0.12133 ± 0.00570 c
200	500	50	408.00 ± 3.05 b	0.15200 ± 0.00251 c	0.15233 ± 0.00233 b
200	500	100	435.00 ± 2.89 a	0.19100 ± 0.00100 a	0.18213 ± 0.00213 a

Mean comparisons were performed employing Duncan’s multiple range test for statistical analysis. The findings are reported as mean values ± standard deviation, derived from three replicate measurements. Columns sharing identical alphabetical superscripts do not differ significantly at *p* ≤ 0.01

Comparison of the mean effects of the three-way salinity stress interaction across varying serotonin and silymarin levels on the SOD and PPO activities revealed that salinity stress without application of these two elicitors significantly increased SOD and PPO activities by 111% and 131%, respectively, compared to normal conditions (Table 2). The highest SOD and PPO activities (0.191 µmol min⁻¹ mg⁻¹ protein and 0.182 µmol min⁻¹ mg⁻¹ protein) were recorded under salinity stress with simultaneous utilization of 100 µM serotonin and 500 µM silymarin. In contrast, the lowest values for these traits (0.0363 µmol min⁻¹ mg⁻¹ protein and 0.0290 µmol min⁻¹ mg⁻¹ protein) were observed under normal conditions (no salinity stress) in the absence of both elicitors (Table 2). Comparison of the dual effects of salinity stress across varying silymarin levels and salinity stress at differing serotonin concentrations revealed that under salinity stress without either elicitor, catalase (CAT), APX, and GPX activities increased significantly compared to normal conditions. Applying 500 µM silymarin under salinity stress elicited 48%, 51%, and 40% increases in CAT, APX, and GPX activities, respectively, relative to silymarin-untreated plants under salinity stress. Similarly, the utilization of 100 µM serotonin under salinity stress enhanced CAT, APX, and GPX activities by 53%, 55%, and 157%, respectively, compared to serotonin-untreated plants under salinity stress (Tables 3 and 4).

**Table 3:** Comparison of the mean effects of the dual interaction between salinity levels and silymarin concentrations on the ascorbate peroxidase, guaiacol peroxidase, and catalase activities

Salinity (mM)	Silymarin concentrations (µM)	Ascorbate peroxidase (µmol min^− 1^ mg^− 1^ protein)	Guaiacol peroxidase (µmol min^− 1^ mg^− 1^ protein)	Catalase (µmol min^− 1^ mg^− 1^ protein)
0	0	0.04333 ± 0.00352 f	0.05222 ± 0.00313 f	0.04955 ± 0.00220 e
0	250	0.04800 ± 0.00358 e	0.05666 ± 0.00377 e	0.05322 ± 0.00256 e
0	500	0.05444 ± 0.00351 d	0.06133 ± 0.00394 d	0.06707 ± 0.00479 d
200	0	0.093666 ± 0.00740 c	0.12122 ± 0.01041 c	0.09877 ± 0.00615 c
200	250	0.10872 ± 0.00830 b	0.14286 ± 0.00929 b	0.11088 ± 0.00668 b
200	500	0.14147 ± 0.00692 a	0.16912 ± 0.00871 a	0.14605 ± 0.00989 a

Mean comparisons were performed employing Duncan’s multiple range test for statistical analysis. The findings are reported as mean values ± standard deviation, derived from three replicate measurements. Columns sharing identical alphabetical superscripts do not differ significantly at *p* ≤ 0.01

**Table 4:** Comparison of the mean effects of the dual interaction between salinity levels and serotonin concentrations on the ascorbate peroxidase, guaiacol peroxidase, and catalase activities

Salinity (mM)	Serotonin concentrations (µM)	Ascorbate peroxidase (µmol min^− 1^ mg^− 1^ protein)	Guaiacol peroxidase (µmol min^− 1^ mg^− 1^ protein)	Catalase (µmol min^− 1^ mg^− 1^ protein)
0	0	0.03711 ± 0.00161f	0.04500 ± 0.00113 f	0.04577 ± 0.00102 f
0	50	0.04733 ± 0.00178 e	0.05566 ± 0.00138 e	0.05688 ± 0.00340 e
0	100	0.06133 ± 0.00163 d	0.06955 ± 0.00193 d	0.06718 ± 0.00380 d
200	0	0.09022 ± 0.00693 c	0.11363 ± 0.00771 c	0.09445 ± 0.00552 c
200	50	0.11288 ± 0.00833 b	0.14158 ± 0.00773 b	0.11 648 ± 0.00739 b
200	100	0.14075 ± 0.00658 a	0.17800 ± 0.00589 a	0.14477 ± 0.00932 a

Mean comparisons were performed employing Duncan’s multiple range test for statistical analysis. The findings are reported as mean values ± standard deviation, derived from three replicate measurements. Columns sharing identical alphabetical superscripts do not differ significantly at *p* ≤ 0.01

### Total phenol and flavonoid content

**Fig. 3:**
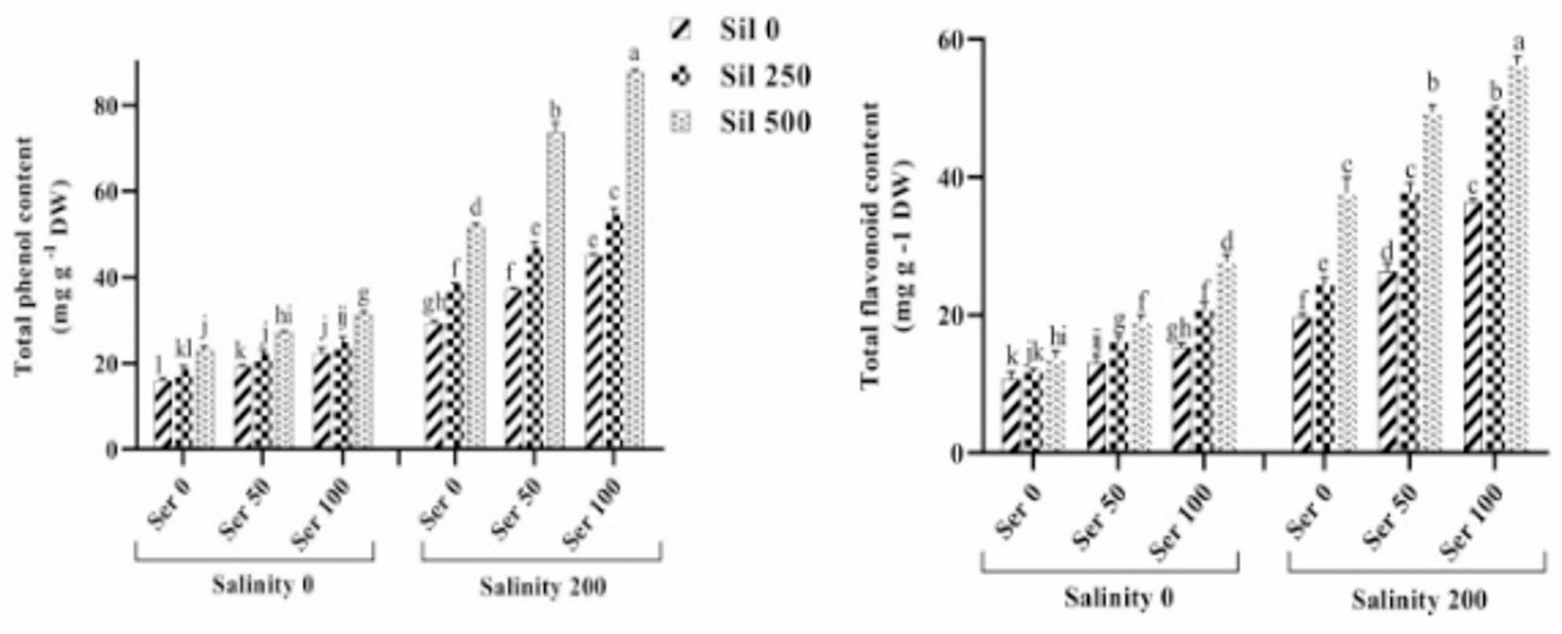
Comparison of the mean triple interaction effects of varying salinity levels (0 and 200 mM), silymarin (0, 250, and 500 µM) (Sil), and serotonin concentrations (0, 50, and 100 µM) (Ser) on the total phenol and flavonoid content. Comparisons of means were performed using Duncan’s multiple range test. Bars sharing identical letters signify no statistically significant differences between treatments

Comparison of the mean effects of the three-way salinity stress interaction across varying serotonin and silymarin levels on the total phenol and flavonoid content revealed that exposure to saline conditions in the absence of both elicitors significantly enhanced the content of these secondary metabolites, with increases of 80% and 84%, respectively, relative to non‑stressed controls. Moreover, combined application of varying serotonin and silymarin concentrations led to a substantial elevation in phenolic and flavonoid levels under both normal and saline conditions when compared to untreated plants grown under the same conditions. Notably, simultaneous treatment with 100 µM serotonin and 500 µM silymarin under salinity stress resulted in pronounced increases of 199% in total phenol content and 186% in total flavonoid content relative to stressed plants that received neither elicitor (Fig. 3).

### Soluble sugar and proline content

**Fig. 4:**
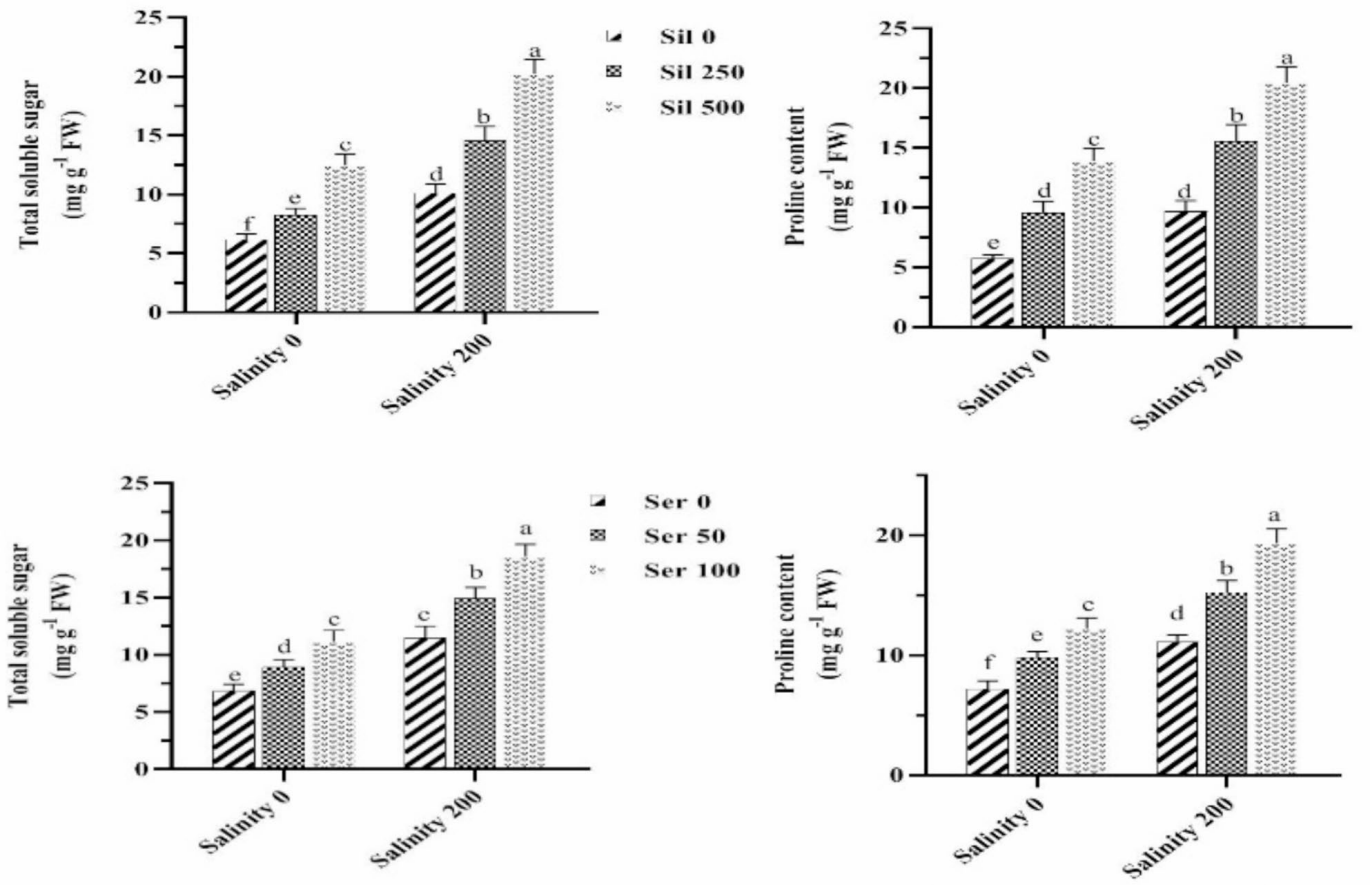
Comparison of the mean dual interaction effects of varying salinity levels (0 and 200 mM) at silymarin (Sil) (0, 250 and 500 µM) and differing salinity levels at serotonin (Ser) concentrations (0, 50, and 100 µM) on the soluble sugar and proline content. Comparisons of means were performed using Duncan’s multiple range test. Bars sharing identical letters signify no statistically significant differences between treatments

Evaluation of salinity stress effects across graded silymarin and serotonin concentrations demonstrated that exposure to saline conditions without either elicitor significantly increased soluble sugar and proline accumulation compared to normal conditions. Under salinity stress, application of 500 µM silymarin elevated soluble sugar and proline content by 100% and 111%, respectively, relative to stressed plants that did not receive silymarin. Likewise, treatment with 100 µM serotonin under saline conditions enhanced these osmolytes by 62% and 74%, respectively, compared to stressed plants cultivated without serotonin (Fig. 4). Notably, among all silymarin treatments applied under salinity stress, the maximum accumulation of soluble sugars (20.22 mg g⁻¹ FW) and proline (20.44 mg g⁻¹ FW) was achieved with 500 µM silymarin. In contrast, the lowest values for both traits (6.16 mg g⁻¹ FW for soluble sugars and 5.72 mg g⁻¹ FW for proline) were recorded in untreated control plants maintained under non‑saline conditions (Fig. 4).

### Mineral elements

**Fig. 5:**
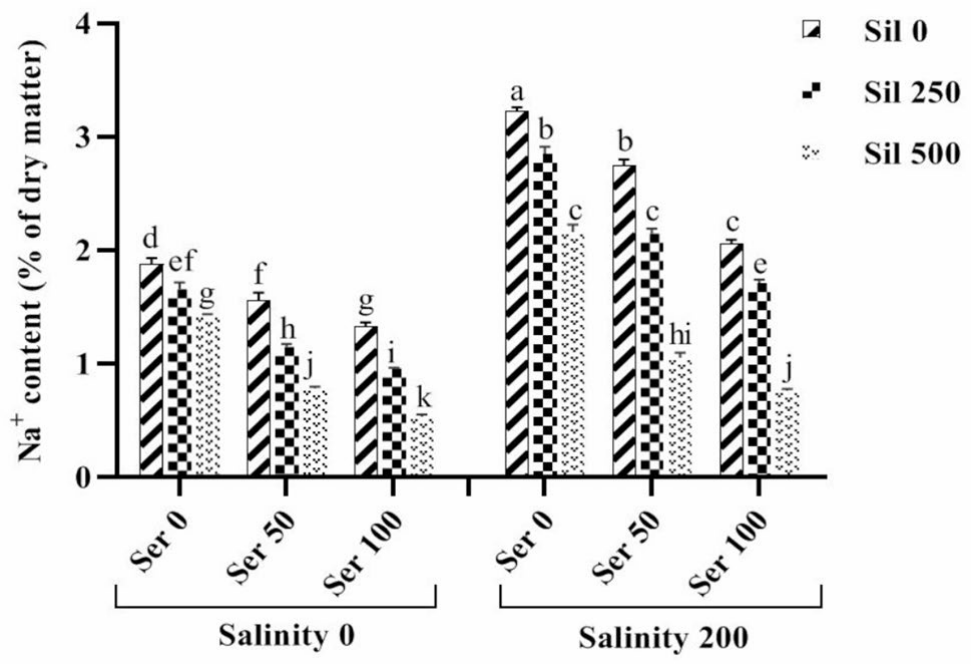
Comparison of the mean triple interaction effects of varying salinity levels (0 and 200 mM), silymarin (0, 250, and 500 µM) (Sil), and serotonin concentrations (0, 50, and 100 µM) (Ser) on the sodium content. Comparisons of means were performed using Duncan’s multiple range test. Bars sharing identical letters signify no statistically significant differences between treatments

**Fig. 6:**
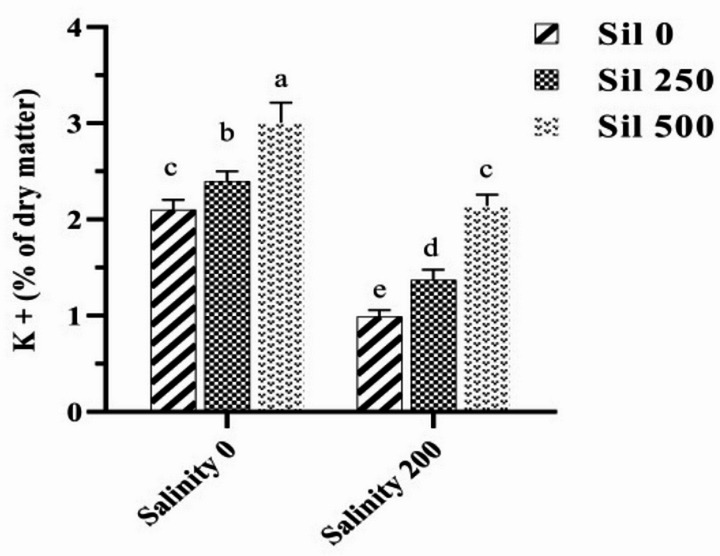
Comparison of the mean dual interaction effects of varying salinity levels (0 and 200 mM) and silymarin (Sil) (0, 250 and 500 µM) on the K^+^ content. Comparisons of means were performed using Duncan’s multiple range test. Bars sharing identical letters signify no statistically significant differences between treatments

A comparative analysis of the average effects of levels of salt stress, in conjunction with varying concentrations of serotonin and silymarin, demonstrated that salt stress, in the absence of these two stimuli, significantly elevated Na^+^ content by 71% relative to normal conditions (Fig. 5). The highest Na^+^ content was recorded under salt stress conditions without the application of either stimulant, measuring 3.23% dry weight. Conversely, the lowest Na^+^ level (0.53% dry weight) was observed in normal conditions, where 100 µM serotonin and 500 µM silymarin were applied. Notably, the simultaneous application of 100 µM serotonin and 500 µM silymarin under salt stress resulted in a substantial 77% reduction in Na^+^ content compared to the conditions lacking these two compounds under salt stress (Fig. 5). A comparative analysis of the average effects of salt stress at varying concentrations of silymarin demonstrated that, under salt stress without the application of these stimulants, K^+^ content significantly decreased by 54% relative to normal conditions. The utilization of 500 µM silymarin under salt stress resulted in a notable increase of 116% in K^+^ content when compared to conditions lacking silymarin under salt stress. Furthermore, the application of different levels of these two stimulants in plants maintained under normal conditions significantly enhanced K^+^ content in comparison to conditions lacking these stimulants (Fig. 6).

### Hydrogen peroxidase and nitric oxide content

**Fig. 7:**
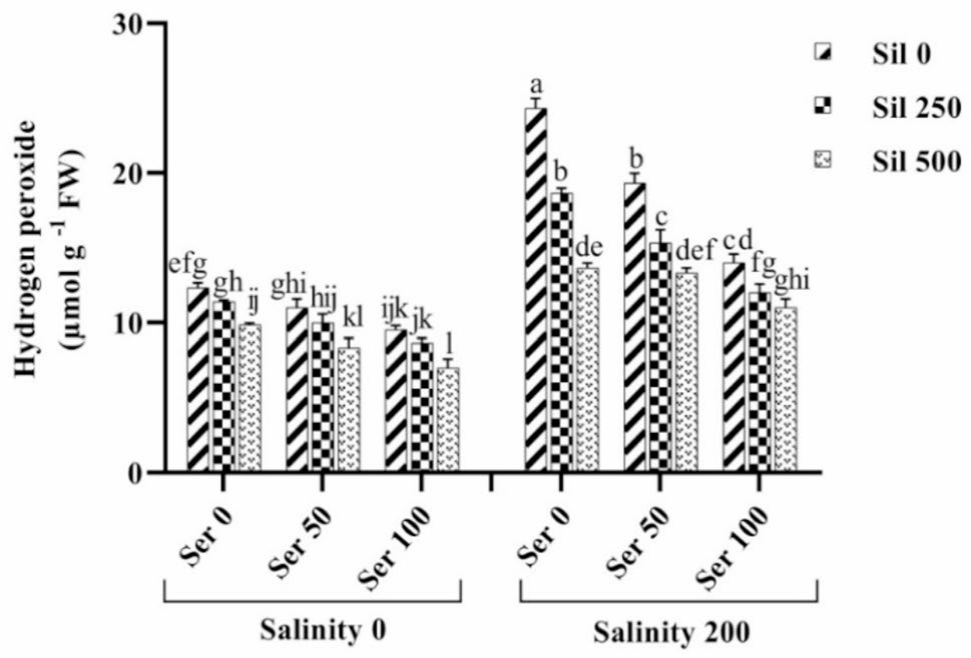
Comparison of the mean triple interaction effects of varying salinity levels (0 and 200 mM), serotonin (Ser) concentrations (0, 50, and 100 µM) and silymarin (Sil) (0, 250, and 500 µM) on the hydrogen peroxidase content. Comparisons of means were performed using Duncan’s multiple range test. Bars sharing identical letters signify no statistically significant differences between treatments

**Fig. 8:**
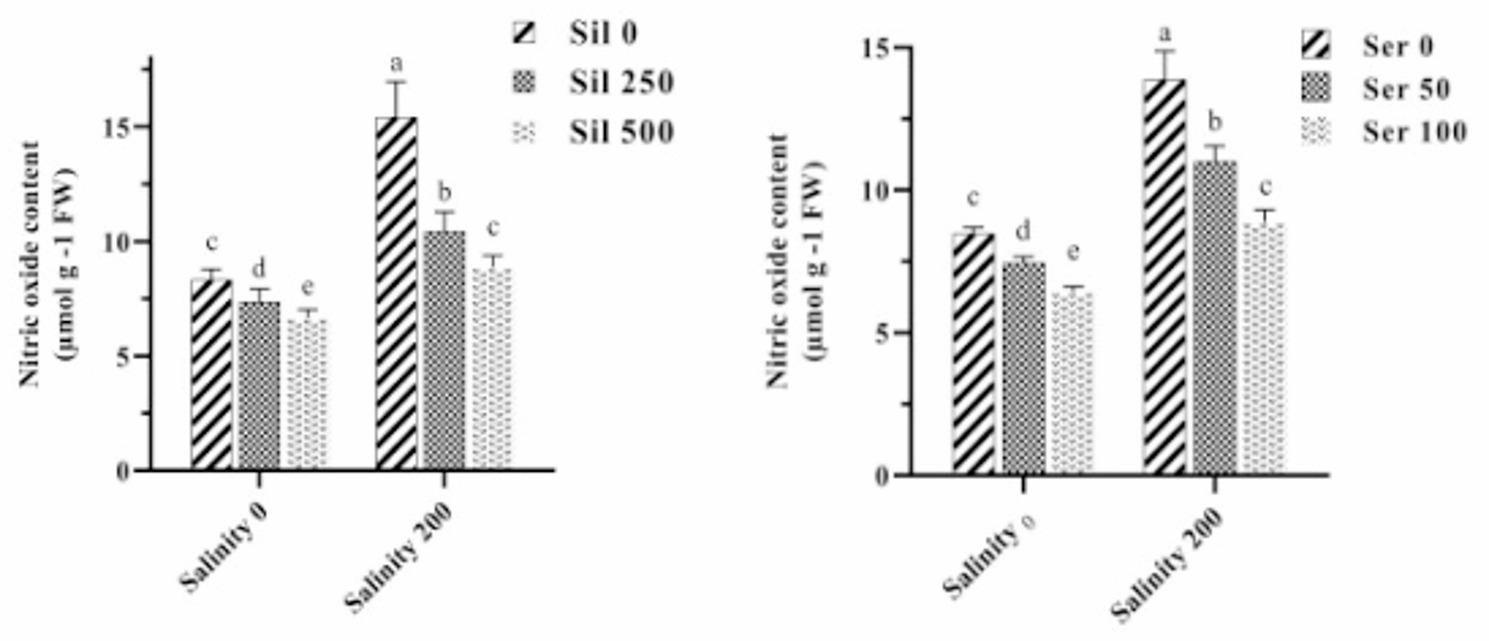
Comparison of the mean dual interaction effects of varying salinity levels (0 and 200 mM) at silymarin (Sil) (0, 250, and 500 µM), and salinity at serotonin (Ser) (0, 50, and 100 µM) on the nitric oxide content. Comparisons of means were performed using Duncan’s multiple range test. Bars sharing identical letters signify no statistically significant differences between treatments

Examination of the combined effects of salinity stress across graded serotonin and silymarin concentrations on H₂O₂ content demonstrated that salinity alone—without supplementation of either compound—significantly elevated H₂O₂ levels by 97% compared to non‑stressed conditions (Fig. 7). The highest H₂O₂ concentration recorded under salinity stress in the absence of both elicitors was 24.33 µmol g⁻¹ FW. Conversely, the lowest H₂O₂ content (7 µmol g⁻¹ FW) was detected under normal conditions following the concurrent application of 100 µM serotonin and 500 µM silymarin. The comparative analysis of the average effects of salt stress at varying concentrations of silymarin and salt stress at serotonin levels on NO content indicated that exposure to saline conditions without either compound significantly increased NO levels by 73% compared to non‑stressed conditions. Application of 250 µM and 500 µM silymarin to plants cultivated under salinity stress resulted in reductions of 29% and 40% in NO content, respectively, relative to stressed plants that did not receive silymarin. Similarly, treatment with 50 µM and 100 µM serotonin under saline conditions led to decreases of 20% and 37% in NO accumulation, respectively, compared to stressed plants maintained without serotonin (Fig. 8).

### Hormone content

**Fig. 9:**
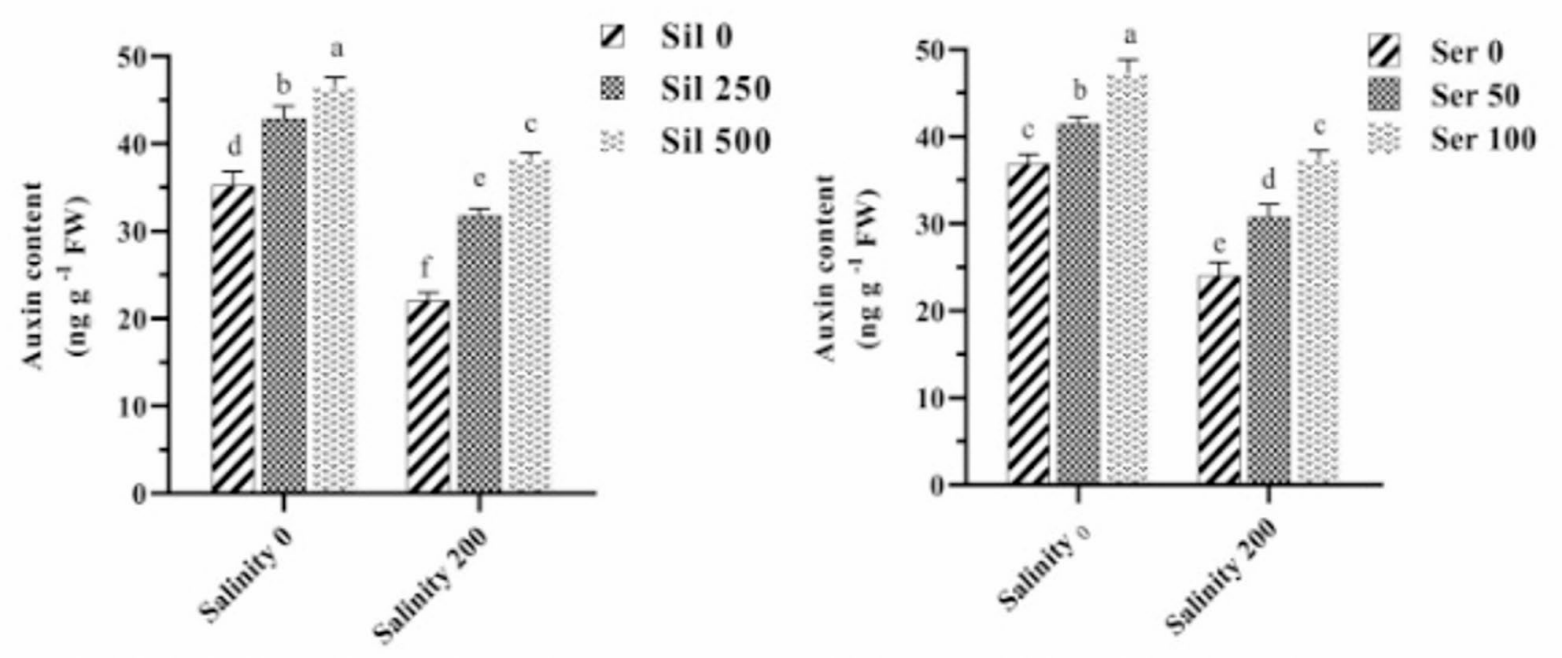
Comparison of the mean dual interaction effects of varying salinity levels (0 and 200 mM) at silymarin (Sil) (0, 250, and 500 µM), and salinity at serotonin (Ser) (0, 50, and 100 µM) on the auxin content. Comparisons of means were performed using Duncan’s multiple range test. Bars sharing identical letters signify no statistically significant differences between treatments

**Fig. 10:**
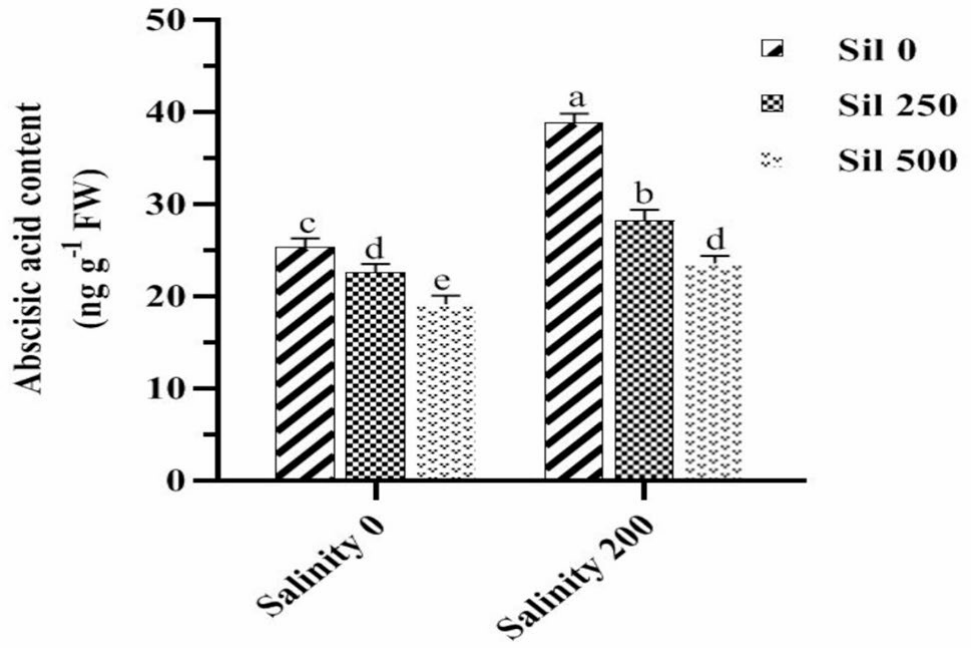
Comparison of the mean dual interaction effects of varying salinity levels (0 and 200 mM) and silymarin (Sil) (0, 250, and 500 µM) on the abscisic acid content. Comparisons of means were performed using Duncan’s multiple range test. Bars sharing identical letters signify no statistically significant differences between treatments

A comparative analysis of the average effects of salt stress at varying concentrations of silymarin and serotonin on the auxin content demonstrated that, under salt stress and in the absence of these stimuli, auxin levels were significantly reduced by 40%, compared to normal conditions. The employment of 250 µM and 500 µM silymarin under salt stress resulted in increases in auxin content of 43% and 73%, respectively, relative to conditions lacking silymarin during salt stress. Furthermore, the utilization of 50 µM and 100 µM serotonin under salt stress led to increases in auxin content of 29% and 57%, respectively, compared to the absence of serotonin under salt stress. Under the combined influence of salinity stress and varying concentrations of silymarin, the highest auxin content (46.55 ng g^− 1^ FW) was recorded with the utilization of 500 µM silymarin (without salinity stress), while the lowest content (21.11 ng g^− 1^ FW) was observed in the absence of silymarin under salinity stress (Fig. 9). Similarly, under the dual influence of salinity stress and varying levels of serotonin, the highest auxin content (47.33 ng g^− 1^ FW) was noted with the application of 100 µM serotonin (without salinity stress), while the lowest content (24 ng g^− 1^ FW) was recorded in the absence of serotonin under salinity stress. A comparative analysis of the average effects of salinity stress at varying concentrations of silymarin on the ABA content indicated that, under conditions of salinity stress and in the absence of silymarin, ABA levels increased significantly by 53%, relative to normal conditions. The maximum (38.77 ng g⁻¹ FW) and minimum (19.01 ng g⁻¹ FW) ABA concentrations were observed under salinity stress treatment conditions (without silymarin application) and the employment of 500 µM silymarin under normal conditions, respectively. Treatment with 250 and 500 µM silymarin in plants cultivated under salinity stress led to a decrease in ABA accumulation of 30% and 45%, respectively, compared to stressed plants maintained without silymarin supplementation (Fig. 10).

### Genes associated with the diosgenin biosynthetic pathway

A comparative analysis of the triple average effects of salinity stress levels, in conjunction with varying concentrations of serotonin and silymarin, on the expression of the *BGL*,* CAS*, and *SQS* genes demonstrated that salinity stress, in the absence of these stimuli, significantly enhanced the expression of these genes by 3.44, 3.66, and 6.66, respectively, relative to normal conditions. The highest levels of expression for the *BGL* (8.15) and *CAS* (10.66) genes were observed under salinity stress conditions with co-application of 100 µM serotonin and 250 µM silymarin. Furthermore, the peak expression of the *SQS* gene (17) was recorded under salinity stress conditions with the simultaneous application of 100 µM serotonin and 500 µM silymarin (Table 5).

**Table 5:** Comparison of the mean triple interaction effects of varying salinity levels, silymarin, and serotonin on the expression of *BGL*,* CAS*, and *SQS* genes

Salinity (mM)	Silymarin concentrations (µM)	Serotonin concentrations (µM)	BGL (Relative expression)	CAS (Relative expression)	SQS (Relative expression)
0	0	0	1.01 ± 0.10 j	1.03 ± 0.06 i	0.99 ± 0.0093 l
0	0	50	2.06 ± 0.66 i	2.16 ± 0.16 h	2.33 ± 0.33 k
0	0	100	3.10 ± 0.10 gh	3.66 ± 0.33 g	3.33 ± 0.33 jk
0	250	0	2.73 ± 0.14 h	2.46 ± 0.29 h	3.06 ± 0.06 jk
0	250	50	3.23 ± 0.12 gh	3.95 ± 0.04 fg	3.66 ± 0.33 j
0	250	100	4.66 ± 0.17 f	5.33 ± 0.33 e	5.33 ± 0.33 i
0	500	0	3.36 ± 0.20 g	3.70 ± 0.15 g	3.66 ± 0.33 j
0	500	50	4.53 ± 0.29 f	4.86 ± 0.13 e	5.33 ± 0.33 i
0	500	100	6.20 ± 0.20 bc	6.90 ± 0.10 cd	7.66 ± 0.66 fgh
200	0	0	3.43 ± 0.29 g	3.66 ± 0.33 g	6.66 ± 0.33 h
200	0	50	4.83 ± 0.16 ef	4.66 ± 0.33 ef	8.66 ± 0.33 ef
200	0	100	6.66 ± 0.33 b	6.66 ± 0.33 d	10.33 ± 0.33 d
200	250	0	4.33 ± 0.33 f	4.90 ± 0.10 e	7.33 ± 0.33 gh
200	250	50	5.86 ± 0.13 cd	7.33 ± 0.33 cd	9.33 ± 0.33 de
200	250	100	8.15 ± 0.31 a	10.66 ± 0.33 a	14.00 ± 0.57 b
200	500	0	5.40 ± 0.20 de	5.33 ± 0.33 e	8.33 ± 0.33 efg
200	500	50	6.66 ± 0.33 b	7.60 ± 0.30 c	12.33 ± 0.66 c
200	500	100	7.90 ± 0.10 a	9.56 ± 0.56 b	17.00 ± 0.57 a

Mean comparisons were performed employing Duncan’s multiple range test for statistical analysis. The findings are reported as mean values ± standard deviation, derived from three replicate measurements. Columns sharing identical alphabetical superscripts do not differ significantly at *p* ≤ 0.01

A comparative assessment of the simple average effects of different levels of salt stress, serotonin, and silymarin on the *SMT* and *SSR* genes expression is illustrated in Table 6. Salinity stress was found to significantly elevate the expression of the *SMT* gene by 1.5 times relative to control conditions. Additionally, treatments involving 250 µM and 500 µM silymarin resulted in increases in *SMT* gene expression by 1.7 and 2.2 times, respectively, compared to untreated plants. Similarly, the application of 50 µM and 100 µM serotonin led to increases in *SMT* gene expression of 1.34 and 2 times, respectively, compared to untreated plants (Table 6). Salinity stress was found to significantly elevate the expression of the *SSR* gene by 1.75-fold compared to control conditions. Moreover, treatment with 250 µM and 500 µM silymarin resulted in increases in *SSR* gene expression of 1.57-fold and 1.83-fold, respectively, compared to untreated plants. Correspondingly, the utilization of 50 µM and 100 µM serotonin led to increases in *SSR* gene expression of 1.5-fold and 1.9-fold, respectively, compared to untreated plants (Table 6).

**Table 6:** Comparison of the mean main interaction effects of varying salinity levels, silymarin, and serotonin on the expression of *SMT*, and *SSR* genes

	SMT (Relative expression)	SSR (Relative expression)
Salinity (mM)		
0	4.68 ± 0.42 b	5.06 ± 0.44 b
200	6.59 ± 0.48 a	8.73 ± 0.50 a
Silymarin concentrations (µM)		
0	3.49 ± 0.42 c	4.78 ± 0.58 c
250	5.92 ± 0.44 b	7.55 ± 0.71 ab
500	7.50 ± 0.50 a	8.36 ± 0.59 a
Serotonin concentrations (µM)		
0	3.75 ± 0.45 c	4.85 ± 0.56 c
50	5.68 ± 0.44 b	6.73 ± 0.54 b
100	7.47 ± 0.54 a	9.11 ± 0.68 a

Mean comparisons were performed employing Duncan’s multiple range test for statistical analysis. The findings are reported as mean values ± standard deviation, derived from three replicate measurements. Columns sharing identical alphabetical superscripts do not differ significantly at *p* ≤ 0.01

**Fig. 11:**
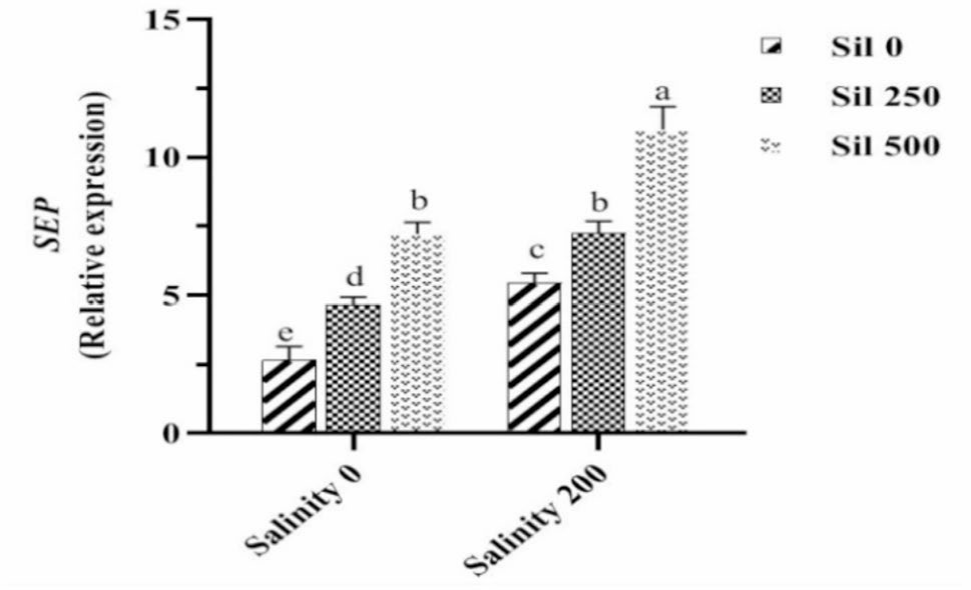
Comparison of the mean dual interaction effects of varying salinity levels (0 and 200 mM) and silymarin (Sil) (0, 250, and 500 µM) on the expression of *SEP* gene. Comparisons of means were performed using Duncan’s multiple range test. Bars sharing identical letters signify no statistically significant differences between treatments

An analysis of the mean dual effects of salinity stress at various concentrations of silymarin revealed that under salinity stress conditions, in the absence of silymarin, the expression of the *SEP* gene significantly increased by twofold compared to normal conditions. The utilization of 250 µM and 500 µM silymarin under salinity stress resulted in increases in *SEP* gene expression of 1.4-fold and 2.1-fold, respectively, relative to conditions without silymarin under salinity stress (Fig. 11).

### Diosgenin content

**Fig. 12:**
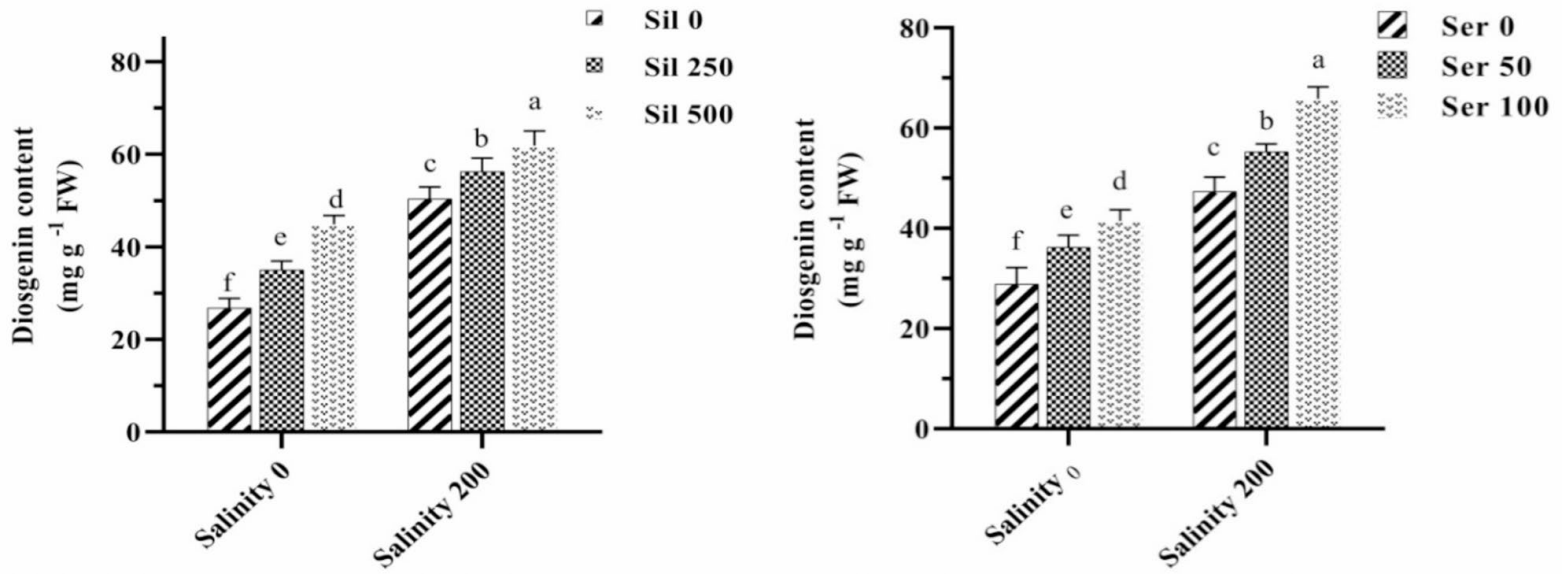
Comparison of the mean dual interaction effects of varying salinity levels (0 and 200 mM) at silymarin (Sil) (0, 250, and 500 µM), and salinity at serotonin (Ser) (0, 50 and 100 µM) on the diosgenin content. Comparisons of means were performed using Duncan’s multiple range test. Bars sharing identical letters signify no statistically significant differences between treatments

Analysis of the combined effects of salinity stress with varying concentrations of silymarin and serotonin on diosgenin accumulation revealed that exposure to saline conditions in the absence of both elicitors significantly elevated diosgenin content by 70% relative to non‑stressed controls. Under salinity stress, application of 250 µM and 500 µM silymarin increased diosgenin levels by 12% and 24%, respectively, compared to stressed plants that did not receive silymarin. Similarly, treatment with 50 µM and 100 µM serotonin under saline conditions enhanced diosgenin content by 18% and 40%, respectively, relative to stressed plants maintained without serotonin supplementation. Notably, application of 500 µM silymarin and 100 µM serotonin resulted in substantial increases of 2.4‑fold and 2.8‑fold in diosgenin accumulation, respectively, in salt‑stressed plants compared to untreated plants under non‑stressed conditions (Fig. 12).

## Discussion

### Pigments level, and relative water content

Significant reductions in all photosynthetic pigments were observed in salinity stress-treated plants compared to the control plants in this study. This decline is likely attributable to disruptions in the biosynthetic enzymes activity involved in pigment production, resulting in either degradation or inhibition of synthesis under stress conditions [25, 26]. These results are consistent with prior research conducted by Liu et al. [27] and Sheikhalipour et al. [28], which reported similar reductions in photosynthetic parameters in the rapeseed and stevia, respectively, under abiotic stress conditions.

In this study, the combined application of silymarin and serotonin was found to synergistically mitigate the adverse effects of salinity stress, thereby enhancing the photosynthetic efficiency of fenugreek plants. The observed improvements in the photosynthetic system of plants treated with silymarin or serotonin may be attributed to reduced in salt uptake and transport within fenugreek cells. The application of 50 µM serotonin has been shown to enhance the activity of photosystem II significantly. Furthermore, it markedly increased the *RbcL* (Ribulose-1,5-bisphosphate carboxylase/oxygenase large subunit) gene expression, which encodes a key enzyme in the Calvin-Benson cycle, thereby improving overall photosynthetic efficiency and energy production and delaying leaf senescence under stress conditions [29]. Serotonin pretreatment significantly upregulates the transcription of the cytochrome P450 monooxygenase gene and the key serotonin biosynthesis gene, tryptophan 5-hydroxylase, under temperature conditions [12]. The findings of this study indicate that fenugreek plants treated with serotonin and silymarin effectively protected their photosynthetic tissues under salt stress. Findings from a recent investigation indicate that the foliar application of silymarin enhances various anatomical features of leaves, particularly stomatal area, length, and width. Furthermore, silymarin contributes significantly to the reinforcement of cell walls and supports sustained cell elongation, thereby improving stomatal structure and preserving chloroplast integrity [30]. This observed enhancement in stomatal traits is likely attributable to silymarin’s role in modulating plant hormones that maintain cell turgor pressure, its influence on the activity of the antioxidant defense system, and its regulatory effect on the differentiation of vascular tissue precursors [31].

The beneficial effects of silymarin and serotonin can be attributed to their capacity to reduce the concentration of salt and other toxic elements, as well as to lower oxidative stress markers, thereby minimizing lipid peroxidation in cell membranes. Collectively, these positive influences contribute to the maintenance of photosynthetic efficiency, likely due to the preservation of relative cell water content [30, 32]. The application of silymarin and serotonin significantly enhanced key photosynthesis indices, including chlorophyll and carotenoid content. This treatment increased the accumulation of photosynthetic products such as sugars and amino acids (e.g., proline), alongside those directly introduced through silymarin application, establishing critical mechanisms for stress tolerance. These augmented photosynthetic compounds contributed to osmotic adjustment, maintenance of cell integrity, elevated RWC, and improved membrane stability index, collectively reducing oxidative stress markers [7, 14, 30, 32].

### Enzymatic and non-enzymatic antioxidants

Our findings indicated that the activities of antioxidant enzymes were significantly elevated in response to salt-induced oxidative stress, with the application of silymarin, and serotonin. This observation aligns with similar trends reported by other researchers [7, 27, 33]. As a free radical scavenger and antioxidant, serotonin, by elevating the activity of antioxidant enzymes, effectively mitigates the detrimental effects of ROS on plants experiencing abiotic stresses [34]. Additionally, serotonin directly regulates the transcriptional levels of antioxidant enzymes, thereby improving plant tolerance to various abiotic stresses [35]. Serotonin also indirectly influences antioxidant defense systems by regulating the activity of other stress-responsive molecules or secondary messengers implicated in ROS signaling [36]. Serotonin engages with specific transcription factors or regulatory elements located within the promoter regions of target genes, thereby modifying their transcriptional activity [37]. While salt stress induced alterations in the physiological and biochemical parameters of fenugreek, the exogenous application of silymarin and serotonin significantly modulated these traits. The enhancement in growth characteristics observed in stressed plants in response to the exogenous application of silymarin or serotonin can be attributed to the accumulation of these compounds, which may play a crucial role in both non-enzymatic and enzymatic antioxidant responses, ultimately protecting plants from oxidative damage [16, 18, 38].

Silymarin enhances the antioxidant defense system through several mechanisms. Firstly, it directly scavenges free radicals. Secondly, it suppresses the activity of particular enzymes involved in the generation of free radicals, thereby preventing their formation and preserving the functionality of the mitochondrial electron transport chain under stress conditions [16]. Thirdly, it plays a role in sustaining the optimal oxidation-reduction state of the cell through stimulation of a variety of antioxidant enzymes and non-enzymatic antioxidants, primarily through transcription factors such as Nrf_2_ (Nuclear factor erythroid 2–related factor 2) and NF-κB (Nuclear factor kappa-light-chain-enhancer of activated B cells) [16, 39]. The application of silymarin significantly reduced concentrations of free radicals, lipid peroxidation, and ELI, thereby improving salinity tolerance in fenugreek plants. The co-application of serotonin and silymarin proved to be a potent strategy for enhancing tolerance of fenugreek under salinity stress by improving photosynthetic capacity. These improvements in photosynthetic efficacy, driven by silymarin, were associated with reduced NaCl accumulation in fenugreek and enhanced antioxidative defense systems, which suppressed oxidative stress markers. Bioactive constituents of silymarin, including flavonoids and polyphenols, demonstrate robust antioxidant properties, efficiently neutralizing ROS, diminishing free radical concentrations, and safeguarding plant tissues against oxidative damage induced by NaCl toxicity [12, 17, 30, 40].

Non-enzymatic antioxidant systems, including phenolics, flavonoids, and anthocyanins, are crucial for maintaining the integrity of photosynthetic tissues under stress conditions [41]. Consistent with previous findings, exposure to salt stress leads to elevated levels of non-enzymatic antioxidants and enhanced enzymatic antioxidant activity, in response to increased H_2_O_2_ levels and ion leakage [42]. In the present study, the application of silymarin and serotonin was found to enhance the accumulation of phenolic compounds and proline, as well as their redox capacities, under both control (non-stressed) and stressed conditions. Previous research has also reported an increase in non-enzymatic antioxidants, including proline, ascorbic acid (AsA), and glutathione (GSH), aimed at suppressing ROS levels in plants externally treated with silymarin under cadmium stress [7, 30]. The enhanced tolerance observed with silymarin application can be attributed to its rich composition of essential organic nutrients, proline, and soluble sugars, which are critical for cellular protoplasm formation [43]. Additionally, silymarin contains phytohormones such as cytokinins and auxins, which drive rapid cell enlargement, division, and multiplication [30, 44]. Previous studies have reported that silymarin enhances plant productivity under stress conditions by accumulating in plants and strengthening their defense mechanisms [45]. These findings align with our results, which underscore silymarin’s role as a potent antioxidant, contributing to increased plant stress resistance. Despite these findings, systematic inquiry remains essential to decipher the precise pathways by which silymarin mediates stress adaptation in plant systems.

### Osmolytes and osmoprotectants

In the present study, plants subjected to salt stress exhibited a significant accumulation of osmolytes, including soluble sugars and proline, compared to those grown under normal conditions. Notably, fenugreek plants treated with silymarin or serotonin under salt stress demonstrated the highest levels of osmolyte accumulation compared to untreated controls, which contributed to enhanced osmotic regulation and provided additional cellular protection against salt stress. The synthesis of these osmolytes is likely a key mechanism through which fenugreek exhibits tolerance to salt stress, as it facilitates osmotic regulation, maintains tissue turgor pressure, and enables growth under severe stress conditions [46]. Functioning as a central metabolic orchestrator, serotonin regulates biosynthetic pathways for compatible solutes and secondary compounds, thereby enhancing cellular adaptation, which is critical for sustaining physiological processes under stress conditions [24]. By improving the accumulation of secondary metabolites, such as phenols and flavonoids, serotonin suppresses free radical release, bolstering plant tolerance to water stress [14]. The role of proline and soluble sugars in enhancing stress tolerance is well-documented across various plant species [47]. Additionally, the results indicate a marked increase in proline content in plants treated with serotonin and silymarin under salt stress. It has been proven that increased proline accumulation following silymarin application was associated with significant modulation of proline metabolism through decreased Δ¹-pyrroline-5-carboxylate synthetase (P5CS) anabolism and increased proline dehydrogenase (ProDH) catabolism, thereby balancing proline concentrations within plant cells [33]. In this study, the application of silymarin optimized photosynthesis, leading to increased accumulation of photosynthetic products such as soluble sugars and free proline, in addition to those introduced through the irrigation of fenugreek plants (bioactive components of silymarin). These compounds contributed to osmotic adjustment, enhanced cell integrity, elevated RWC, and improved membrane stability index. Overall, the findings of this study indicate that the ameliorative effect of silymarin application mitigated the adverse impacts of salt stress on fenugreek plants. This protective role was achieved through the upregulation of osmoregulatory compounds and various components of the antioxidant defense system, thereby facilitating the scavenging of reactive oxygen species and maintaining cellular osmotic balance [30, 48].

### Membrane peroxidation, hydrogen peroxide, and nitric oxide

Exposure to environmental stressors such as salinity or drought triggers excessive generation of reactive oxygen species, among which H₂O₂ plays a prominent role [49]. In parallel, NO operates as a critical signaling agent, engaging in extensive crosstalk with H₂O₂ throughout the plant’s adaptive response to adverse conditions. This interaction manifests at various levels of cellular regulation, encompassing biosynthetic pathways and transcriptional control mechanisms that together integrate stress-related signals and coordinate defensive strategies [50]. Under abiotic stresses, an initial surge in NO and H₂O₂ production serves as an early signaling event; however, when sustained, this elevation results in unchecked oxidative damage evidenced by increased MDA from lipid peroxidation [51]. Treatment with silymarin and serotonin restores cellular equilibrium through a multifaceted mode of action: first, by directly scavenging reactive oxygen species and thereby limiting H₂O₂ accumulation; second, by upregulating both enzymatic and non-enzymatic antioxidant defenses; third, by modulating NO signaling pathways to prevent nitrosative stress; and fourth, by consequently reducing membrane lipid peroxidation, reflected in diminished MDA levels. This integrated antioxidant intervention effectively interrupts the cycle of oxidative deterioration, safeguards membrane integrity, and enhances overall plant performance under stress conditions [10, 24, 27, 30, 52, 53].

In the case of serotonin, current understanding indicates that its capacity to modulate NO signaling is largely mediated by melatonin, a secondary metabolite derived directly from serotonin through enzymatic conversion. Serotonin constitutes the immediate biochemical precursor in the melatonin biosynthesis pathway, and a well-characterized regulatory interplay between melatonin and NO has been firmly established in the context of plant responses to abiotic stress. Melatonin signaling is closely linked with the regulation of nitrogen metabolism and nitrosative stress, with NO acting as a downstream component in the signal transduction cascades triggered by melatonin [49]. Of particular significance is the coordinated action of melatonin and NO in modulating the activity of antioxidant enzymes, maintaining cellular homeostasis of reactive oxygen and nitrogen species, and regulating glutathione metabolism—all of which collectively contribute to the preservation of redox balance under adverse environmental conditions. The interaction between melatonin and NO is embedded within complex signaling networks that integrate calcium-dependent pathways, stress-responsive transcriptional regulators, and post-translational protein modifications. Through this indirect route, serotonin contributes to NO signaling by supplying the biosynthetic substrate necessary for melatonin production, thereby influencing the pool of melatonin available for stress adaptation responses [54, 55].

Regarding silymarin, although direct evidence at the molecular level in plant systems remains relatively scarce, studies conducted on milk thistle—the plant species from which silymarin is naturally derived—offer instructive findings concerning the relationship between NO and silymarin accumulation [52, 56]. Application of sodium nitroprusside, a well-characterized NO donor, under water deficit conditions was shown to significantly increase the concentration of multiple silymarin constituents, including taxifolin, silychristin, silybin B, and isosilybin B. This enhancement in silymarin accumulation coincided with a marked reduction in oxidative stress indicators such as malondialdehyde and hydrogen peroxide. Further observations revealed that NO treatment contributed to improved photosynthetic performance, increased pigment content, and enhanced seed yield in drought-exposed milk thistle plants. Collectively, these findings support the view that NO exerts a positive regulatory influence on silymarin biosynthesis and accumulation, likely through its effects on cellular oxidative status and the modulation of secondary metabolic pathways [52, 56]. The capacity of NO to mitigate oxidative damage induced by water deficit further reinforces the existence of a functional relationship in which NO promotes the production of silymarin, which in turn contributes to stress tolerance through its well-documented antioxidant properties. In summary, while serotonin interfaces with the NO signaling network indirectly via melatonin, silymarin accumulation appears to be upregulated by NO under conditions of environmental stress, suggesting a bidirectional interaction that warrants further detailed investigation at the mechanistic level [52, 56].

### Hormonal regulation

Salt stress alters auxin content and its threshold levels in plants. Under abiotic stress conditions, the inhibition of auxin biosynthesis is likely to lead to an accumulation of serotonin in plant tissues [29]. Serotonin influences both auxin biosynthesis and transport in plants. Serotonin enhanced salt tolerance in fenugreek by positively regulating the ABA biosynthesis pathway. Moreover, serotonin-pretreated fenugreek seedlings exhibited elevated ABA levels under normal conditions. Consequently, the enhanced salinity tolerance observed in these seedlings may be attributed to the serotonin-mediated stimulation of endogenous ABA synthesis under salt stress conditions [57, 58]. In the current research, exposure to salt stress resulted in a notable reduction in auxin concentration and an increase in ABA levels in fenugreek relative to control plants. Conversely, the administration of different concentrations of serotonin and silymarin effectively elevated auxin content while decreasing ABA content under both salt stress and normal conditions, relative to untreated plants.

Exogenous serotonin application markedly elevates zatin (a cytokinin derivative) and ABA biosynthesis, indicating its collaborative interplay with cytokinin and ABA pathways in regulating essential morphogenetic processes [59]. These findings underscore the multifaceted role of serotonin in modulating hormonal interactions that influence plant development and stress responses. Serotonin is crucial for regulating the expression of enzymes associated with various phytohormones, transcripts, and cofactors that are integral to signaling interactions [49, 59, 60]. Overall, serotonin orchestrates fundamental growth trajectories and physiological responses to abiotic challenges across plant species [60]. The bolstered antioxidant defense system in fenugreek plants treated with serotonin-silymarin effectively reduced Na + ion accumulation, facilitating stress recovery through the suppression of ROS. This, in turn, likely enabled plants to stabilize hormonal balance. Our study revealed that the application of silymarin, or serotonin, significantly elevated the hormonal content of auxin. This increase was associated with enhanced activity of the antioxidative defense system, alongside a marked reduction in ROS, including H₂O₂. These effects collectively minimized MDA levels, ELI, and NaCl accumulation, while optimizing plant performance and photosynthetic efficiency.

The findings of a recent investigation on faba bean provide additional support for the observations made in the present study. In that work, which examined the effects of biostimulant formulations containing honey, iodine, and silymarin under drought stress conditions, foliar application of a silymarin-enriched treatment led to substantial increases in the levels of growth-promoting hormones, including IAA (Indole-3-acetic acid), GA_3_ (Gibberellic acid ), and cytokinins, while concurrently reducing ABA accumulation. Specifically, plants subjected to the silymarin-containing formulation exhibited IAA content elevated by 71%, GA₃ by 73%, and total cytokinins by 86%, alongside a 45% decline in ABA levels relative to untreated controls subjected to drought [30]. This pattern of hormonal adjustment, characterized by the upregulation of hormones associated with growth promotion and the downregulation of the primary stress hormone ABA, closely parallels the results obtained in the current study. Such convergence across studies involving different abiotic stress conditions suggests that silymarin may exert a conserved function in re-establishing hormonal equilibrium when plants face environmental challenges [30, 40].

Several mechanisms, grounded in existing literature, may account for the hormonal adjustments observed following silymarin application. One prominent factor is the well-documented antioxidant capacity of silymarin, which mitigates oxidative stress by reducing the accumulation of reactive oxygen species, hydrogen peroxide, and malondialdehyde. This reduction in oxidative damage helps preserve cellular integrity and creates a stable environment conducive to proper hormone synthesis and signaling [7, 15]. Additionally, silymarin has been shown to contain endogenous phytohormones, including IAA, GA₃, and cytokinins, which could contribute directly to the total hormone pool available to the plant. Beyond its direct hormonal content, silymarin also influences membrane dynamics by preventing lipid peroxidation through its radical-scavenging activity, enhancing intracellular glutathione levels, and modulating membrane permeability [30, 40]. Its ability to maintain membrane fluidity via direct interaction with lipid components further supports optimal membrane function. The silibinin fraction of silymarin, in particular, acts as an effective chelator of ferrous ions, thereby inhibiting Fenton-type reactions that generate highly reactive hydroxyl radicals and protecting cellular structures from oxidative injury. Collectively, these membrane-stabilizing actions are critical for sustaining hormone receptor activity and the downstream signal transduction pathways upon which hormonal responses depend [30, 40].

When applied together, serotonin and silymarin appear to operate through complementary pathways that reinforce one another. Serotonin is recognized as a central integrator of multiple stress signaling cascades, including those mediated by phytohormones, calcium fluxes, reactive oxygen species, and stress-responsive transcriptional networks. Silymarin complements this regulatory role by furnishing robust antioxidant protection and contributing directly to the maintenance of hormonal equilibrium. Through the combined suppression of oxidative stress, preservation of photosynthetic function, and fine-tuning of the balance between auxin and ABA, this synergistic interaction ultimately enhances plant performance under saline conditions, as reflected in the experimental data presented here [7, 15, 30, 40]. The alignment between the outcomes of the faba bean study and those of the current investigation lends considerable weight to the conclusion that silymarin, particularly when integrated with other bioactive constituents, functions as an effective modulator of hormonal activity under adverse environmental conditions. Both studies demonstrate a consistent capacity of silymarin to elevate levels of growth-associated hormones while diminishing the accumulation of ABA, thereby reinforcing plant tolerance to abiotic stress. These observations highlight the diverse functional roles of silymarin in regulating hormonal networks that govern plant development and adaptive responses to stress.

### Ionic balance

Our findings indicated that salt stress leads to an increase in Na^+^ ion accumulation while concurrently decreasing K^+^ ion levels in fenugreek. Notably, treatment with varying concentrations of silymarin and serotonin resulted in K^+^ levels in the leaves remaining significantly higher than those observed in untreated plants. This suggests that the development of plant resistance can be enhanced through effective ion uptake mechanisms under both normal and salt stress conditions. The beneficial effects of silymarin and serotonin on ion uptake, coupled with their inhibitory effects on Na^+^ uptake, may contribute to the capacity of fenugreek seedlings to tolerate high salinity levels under salt stress [7, 61]. Additionally, serotonin has been shown to regulate the transport of specific ions across biological membranes in plants, further supporting the notion that serotonin application can alleviate salt stress [61, 62]. Collectively, these findings underscore the efficacy of serotonin and silymarin in regulating ion homeostasis and bolstering plant tolerance to salinity stress. The protective mechanisms of serotonin may include the detoxification of salt-induced ROS and the regulation of osmotic pressure through the modulation of ion mobility within the roots [37]. Additionally, the growth and morphology of roots may be influenced under salt stress conditions via complex biochemical interactions involving melatonin, NO, and auxin [63]. This study’s results indicate that the application of silymarin and serotonin was the most effective treatment for mitigating salinity stress in fenugreek plants. Silymarin and serotonin significantly alleviated damage to fenugreek plants by enhancing the K⁺/Na⁺ ratio (through regulating ion channels, transporters, and pumps responsible for the uptake, efflux, and compartmentalization of ions such as K⁺ and Na⁺), non-enzymatic antioxidant and osmoprotectant levels, enzymatic antioxidant activities, and phytohormone contents, while reducing Na⁺ accumulation, oxidative stress markers, ABA levels, and oxidative stress-induced membrane damage [64, 65]. These beneficial effects are attributed to the bioactive components of silymarin. Consequently, silymarin represents a cost-effective biostimulant and micronutrient source for plants, offering a viable alternative to costly synthetic compounds [7]. Rigorous dissection of silymarin’s polyvalent actions—particularly its integration of stress perception, signaling networks, and phenotypic adaptations—represents an essential research frontier.

### Diosgenin content

Current research has established that water deficit conditions lead to increased levels of total phenolic and total flavonoid content in various plant species [66]. This suggests that strategic management of environmental stressors could be utilized to optimize the synthesis of essential secondary metabolites in plants. Research findings consistently demonstrate that diosgenin levels are significantly impacted by various treatments, including cold plasma, the combined application of melatonin with salinity stress and cold plasma, titanium dioxide under low-temperature stress, and epibrassinolide under high-temperature stress [3, 5, 67–69]. The precise mechanisms by which serotonin upregulates various secondary metabolites via interactions with different metabolic pathways, including those involving growth regulators, remain inadequately understood. Total phenolic content has been shown to significantly increase following serotonin treatment [14]. This enhancement is believed to occur through the interaction of serotonin with the phenylpropanoid pathway [70]. Previous studies have documented that the application of serotonin and NaCl, both in ex vitro and in vitro plant systems exposed to abiotic or biotic stress conditions, initiates a cascade of intracellular signaling pathways. These pathways induce biochemical and molecular changes [71].

The adoption of innovative approaches, including the use of diverse stimuli and the exploration of their impacts on the expression and regulation of biosynthetic pathways, marks a significant step forward in the commercial production of valuable secondary metabolites [72]. In the current study, the treatment of fenugreek plants with salt, serotonin and silymarin stimuli significantly enhanced the expression of genes implicated in the biosynthesis of diosgenin, as well as the overall diosgenin content. The results of this investigation underscore the efficacy of employing a combined approach of abiotic and biotic stimuli as a rapid, effective, and environmentally sustainable method for increasing diosgenin levels. Various strategies, including agricultural practices (such as fertilization), laboratory techniques (such as tissue culture), and genetic approaches (including gene transfer), have been proposed by researchers to augment the content of secondary metabolites in plants [73]. However, consensus on the optimal method has been elusive, primarily due to concerns regarding environmental pollution, time-intensive processes, high costs, and ethical considerations associated with the generation of transgenic plants. In contrast, the utilization of biotic stimuli is gaining traction owing to their cost-effectiveness, safety (as they are naturally part of the plant’s structure), and the rapid outcomes they produce [72]. Nevertheless, applying stimuli to enhance secondary metabolite production is not without its challenges. Critical factors must be considered, including the specific plant species or genotype employed, the nature of the abiotic stressor, the concentration and type of stimulus used, as well as the age and growth stage of the plant [74]. These considerations are essential for optimizing the conditions under which secondary metabolite accumulation can be maximized.

## Conclusion

The findings of the present study highlight a promising strategy for the treatment of plants with serotonin and silymarin under conditions of salt stress. Following the treatment of fenugreek plants with silymarin and serotonin, there was a notable increase in the content of photosynthetic pigments, specifically chlorophylls and carotenoids, along with improvements in various physiological and biochemical traits. These enhancements may be attributed to the physiological roles of silymarin and serotonin in modulating defense responses to salt stress through multiple protective mechanisms, including the regulation of genes associated with defense mechanisms and phytohormones. These enhancements contributed to the mitigation of lipid peroxidation, reduced ion leakage, and minimized oxidative damage induced by ROS. The successful outcomes of this treatment can be attributed to the effective limitation of Na^+^ ion accumulation, alongside the activation of the antioxidant defense mechanisms in response to salt stress. Consequently, the results of this study advocate for the application of silymarin and serotonin as innovative and effective biostimulants that can promote various physiological and metabolic processes, thereby increasing stress tolerance in fenugreek plants. Capitalizing on serotonin’s master regulatory capacity, dual strategies of metabolic engineering for elevated endogenous levels and field-applicable serotonin/precursor formulations present transformative opportunities for fortifying crop stress tolerance and establishing regenerative farming practices.

## Methods

### Methodology and experimental conditions

Plant material was collected under the supervision and authorization of the Ministry of Agriculture Jihad in Tehran, Iran, in strict adherence to national and local regulations. All authors guaranteed complete adherence to the established local and national guidelines. The study treatments consisted of salinity stress at 200 mM, combined with varying concentrations of serotonin (0, 50, and 100 µM) and silymarin (0, 250, and 500 µM). Every combination of treatments was administered to three separate pots, representing three replicates, with each pot containing five disinfected fenugreek seeds (Boshruyeh genotype). The pots, each weighing approximately 3 kg, were filled with a homogeneous mixture of peat moss, perlite, and field soil in equal proportions. Stock solutions of serotonin and silymarin, each at a concentration of 10,000 µM, were prepared by dissolving the respective growth stimulants in ethanol. These solutions were stored at 4 °C in dark conditions to maintain stability. This experiment was carried out in a growth chamber from June to July 2024, with the period from planting to leaf harvest lasting approximately six weeks. Disinfected fenugreek seeds were sown in the prepared pots and irrigated approximately every four days. At the four-true-leaf stage, to enhance plant resilience, the seedlings were treated with the designated concentrations of serotonin and silymarin solutions. Each pot was uniformly treated with 150 mL of the selected solutions for seven days. Subsequently, to induce salinity stress, plants were irrigated with 150 mL of a 200 mM sodium chloride solution for an additional seven days. The experiment was conducted in a controlled growth chamber maintained at 24 °C during the day and 22 °C at night, with a photoperiod of 16 h of light and 8 h of darkness. Relative humidity was set at 60–65%. At six weeks post-sowing, coinciding with the onset of flowering, leaf samples were collected and immediately frozen at -80 °C for subsequent analysis.

### Chlorophyll and carotenoid quantification

The chlorophyll and carotenoid content were determined using a UV-1800 spectrophotometer (Shimadzu, Japan), following the protocol outlined by Arnon [75]. Absorbance measurements at 480, 645, and 663 nm were transformed into pigment concentrations.

### Relative water content assessment

To assess RWC, five healthy leaves of uniform size were selected from each treatment group and evaluated using the method and formula established by Kirnak et al. [76].

### Electrolyte leakage index measurement

To determine the ELI, five leaf discs of uniform size were excised from the leaves for each treatment using a standardized punch. Subsequent steps were conducted in conformance with the established methodology by Hepburn et al. [77].

### Malondialdehyde content assessment

To evaluate MDA content, the steps were conducted following the methodology outlined by Stewart and Bewley [78]. Ultimately, the specimensꞌ absorbance was determined employing a spectrophotometer at wavelengths of 532 nm and 600 nm.

### Total soluble protein content and antioxidant enzyme activity assessment

Total soluble protein content was quantified using the method established by Bradford [79]. Subsequently, the absorbance of supernatant was measured at 595 nm using a spectrophotometer. Superoxide dismutase activity was evaluated using the method described by Acar et al. [80], which measures the suppression of the photochemical reduction of nitroblue tetrazolium at a wavelength of 560 nm. Catalase activity was assessed using the method established by Aebi [81], which employs H_2_O_2_ as the substrate. The change in absorbance was measured at a wavelength of 240 nm over a period of 5 min. Ascorbate peroxidase activity was evaluated using the method described by Madhusudhan et al. [82]. The activity of APX was assessed by monitoring the reduction in absorbance at 290 nm, indicative of ascorbic acid oxidation. Guaiacol peroxidase activity was assessed using the methodology established by Azevedo Neto et al. [83]. The formation of tetraguaiacol was monitored by measuring the absorbance at a wavelength of 470 nm over a period of 5 min. The activity of PPO was assessed following the protocol outlined by Ogel et al. [84], utilizing pyrocatechol as the substrate. The absorbance of the O-quinone product was determined at a wavelength of 420 nm.

### Total phenol content assay

The total phenol content was evaluated using the Folin-Ciocalteu assay, as described by Lamuela-Raventós [85], with the absorbance of the supernatant determined at a wavelength of 765 nm.

### Total flavonoid content assay

The total flavonoid concentration was assessed employing the aluminum chloride colorimetric assay, as described by Quettier-Deleu [86]. The optical density of the supernatant was measured at a wavelength of 415 nm.

### Determination of soluble sugar content

To assess the soluble sugar content, 250 mg of crushed leaf samples were completely blended with 2 mL of 80% ethanol and homogenized. Finally, the optical density of the supernatant was determined at a wavelength of 485 nm to assess the concentration of soluble sugar, as described by Omokolo et al. [87].

#### Quantification of proline content

To determine proline content, 250 mg of crushed leaf tissue was combined with 4 mL of 3% sulfosalicylic acid and homogenized. Subsequently, the absorbance of the upper supernatant was registered at 520 nm, as described by Bates et al. [88], to quantify the proline content.

#### Potassium and sodium content

For the assessment of K^+^ and Na^+^ content, leaf tissue from each treatment was dried in an oven at 75 °C for 48 h. Subsequently, 0.5 g of the leaf tissue was mixed with 10 mL of 0.1 N acetic acid and vortexed thoroughly. Finally, the concentrations of K^+^ and Na^+^ in the extracts were measured using a flame photometer, as described by Isaac and Kerber [89].

### Auxin content

To quantify auxin content, a mixture of 20 mL of methanol and distilled water in equal proportions was combined with 1.5 g of leaf tissue. Other extraction steps were performed according to the protocol described by Dobrev and Vankova [90]. The analysis was conducted utilizing a C18 high-performance liquid chromatography (HPLC) column, with a particle size of 4.6 μm, a length of 25 cm, and an internal diameter of 5 mm. The flow rate was kept at 0.8 mL/min, with the mobile phase consisting of a gradient blend of formic acid and acetonitrile. The gradient specifications were as follows: from 0 to 5 min, the composition was 90:10% formic acid to acetonitrile. The transition from 95:5% to 0:100% formic acid to acetonitrile occurred between 5 and 6 min, and from 6 to 16 min, the composition remained at 0:100% formic acid to acetonitrile. To measure the auxin concentration, a linear regression analysis was performed based on the standard calibration curve, employing various auxin concentrations at a detection wavelength of 254 nm (Supplementary Fig. 1).

### Abscisic acid content analysis

To quantify the ABA content, 2 g of leaf tissue were combined with 3 mL of an extraction solution composed of methanol, ethyl acetate, and acetic acid in a volume ratio of 1:50:49. Other extraction steps were performed according to the protocol described by Hubick and Reid [91]. The quantification of ABA was performed using HPLC from Agilent Technologies, USA. The flow rate was sustained at 0.8 mL/min, with the detection wavelength established at 254 nm. The mobile phase for the HPLC analysis was made up of two separate components. Component “A” was prepared using water, acetonitrile, and formic acid in a volume ratio of 94.9:5:0.1, whereas component “B” consisted of the mentioned solutions in a ratio of 89.9:10:0.1, as outlined by Dobrev and Vankova [90]. The quantification of ABA concentration was accomplished using standard ABA, which yielded a correlation coefficient (R²) of 0.989 for the evaluated concentrations (Supplementary Fig. 1).

### Diosgenin content analysis

To assess the diosgenin content, powdered leaf tissue (1 g) was homogenized with 20 mL of 96% ethanol and subjected to ultrasonic treatment for 30 min at room temperature. Other extraction steps were performed according to the protocol described by Zolfaghari et al. [92]. The HPLC column utilized was a C18 type with a particle size of 4.6 μm, a length of 25 cm, and an internal diameter of 5 mm. The flow rate was maintained at 0.8 mL/min, with the detection wavelength set at 210 nm. The mobile phase was composed of a blend of acetonitrile and water in a ratio of 90:10. To quantify the endogenous diosgenin content, a linear regression analysis of the standard calibration curve was established using diosgenin standards at varying concentrations (100, 150, 300, 500, and 1000 ppm) (Supplementary Fig. 2). The injection volume for the HPLC analysis was 20 µL, and the R^²^ for the data obtained was 0.99.

#### Nitric oxide content analysis

Following the spectrophotometric protocol of Zhou et al. [93], NO levels were assessed through absorbance readings of reaction products at 540 nm.

### Hydrogen peroxide content analysis

The assessment of H_2_O_2_ content was conducted using a reaction mixture comprising 5 mL of 0.1% trichloroacetic acid and 0.5 g of powdered leaf tissue. The optical density of the sample was assessed at 390 nm, and the H_2_O_2_ concentration was calculated by referencing a standard calibration curve constructed with varying H_2_O_2_ concentrations [94].

#### RNA extraction and cDNA synthesis

Total RNA was isolated utilizing the RNeasy Plant Mini Kit (Qiagen), following the manufacturer’s protocol. The integrity and concentration of the extracted RNA were assessed through Nanodrop spectrophotometry and 1% agarose gel electrophoresis. cDNA synthesis was performed using the Thermo Scientific RevertAid Strand cDNA synthesis. Primers designed to evaluate the expression profiles of target genes were sourced from previous studies [87]. The characteristics of these primers utilized in the current study are summarized in the Supplementary Table 2.

### Real-time PCR reaction

In the gene expression analysis, real-time PCR was conducted using the RealQ Plus 2x Master Mix Green, adhering to the manufacturer’s guidelines. Reactions were assembled in a 20 µL total volume, constituted by 10 µL master mix, 2 µL of primers, 2 µL of cDNA, and 6 µL of nuclease-free water. Each experiment included three biological and two technical replicates to ensure the reliability of results. The qPCR amplification protocol was configured for 35 cycles, with denaturation at 95 °C (20 s) followed by primer annealing at 60–61 °C (40 s) per cycle using an ABI Stepone thermocycler.

### Statistical analyses

Statistical significance was evaluated using analysis of variance (ANOVA), followed by Duncan’s multiple range test (*p* < 0.01) for comparing means, performed in SPSS version 26. Real-time PCR data were analyzed using the Livak method, applying the 2^^−ΔΔCt^ formula as outlined by Livak and Schmittgen [95].

## Supplementary Information

Supplementary Material 1.

Supplementary Material 2.

## Data Availability

The data generated or analyzed in this study are included in this article. Other materials that support the findings of this study are available from the corresponding author on reasonable request.
