# Supplementary material for: Silymarin and serotonin, individually and synergistically, enhance fenugreek resistance to salt stress by modulating pathways involved in chlorophyll biosynthesis, defense system, hormonal regulation, ion redox balance, and diosgenin production
Source: BMC Plant Biol. 2026 Apr 17;26:929. doi: 10.1186/s12870-026-08765-7 (PMC13224543; doi:10.1186/s12870-026-08765-7)
Supplement: Supplementary file 1 — Supplementary Material 1. [file 12870_2026_8765_MOESM1_ESM.docx]

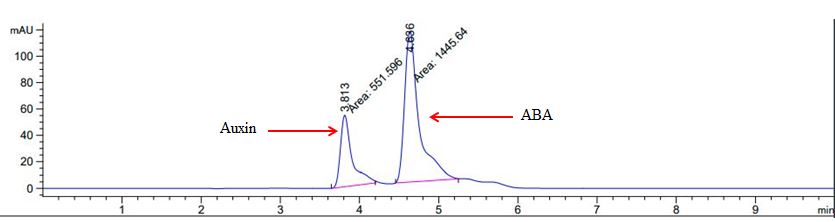


**Figure 1.** The HPLC chromatogram illustrates the mixture of standard Auxin and ABA, each with a concentration of 200 ppm.

**B**

**A**


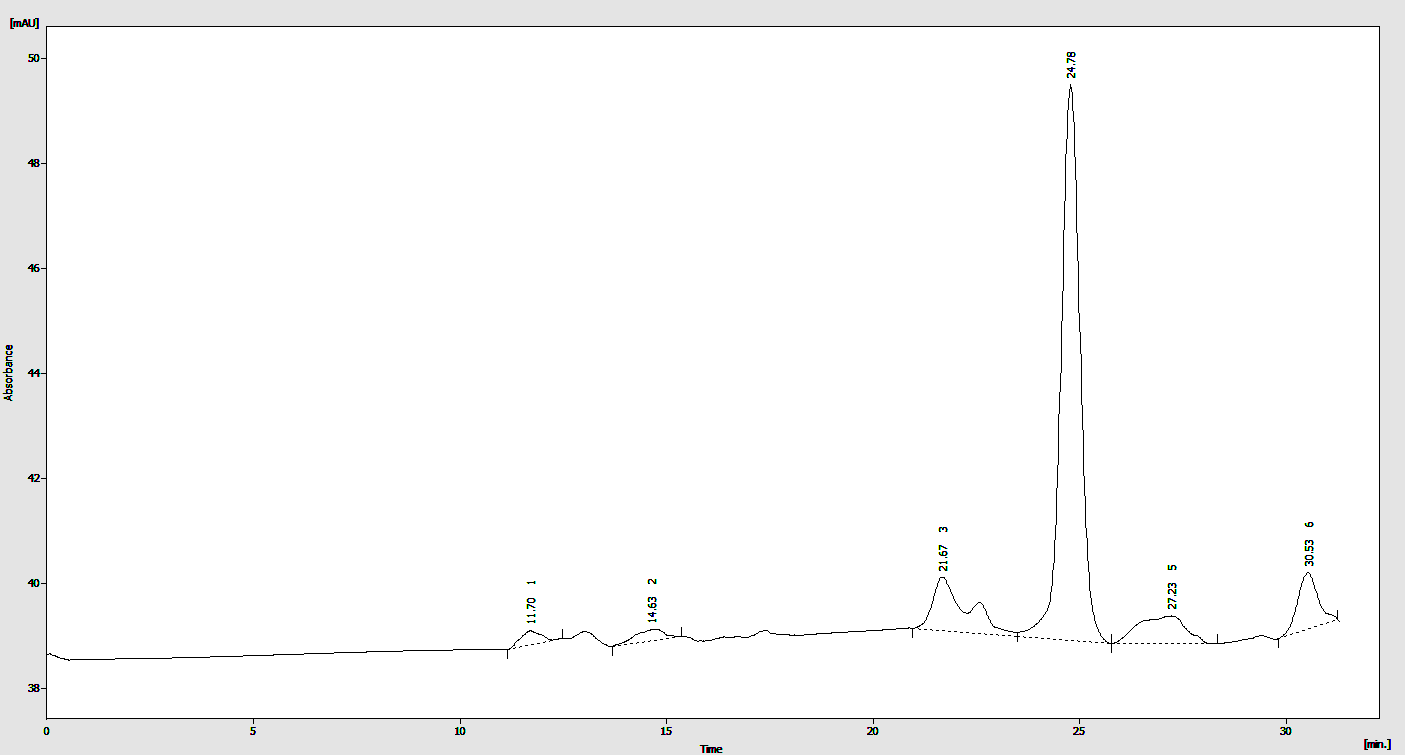

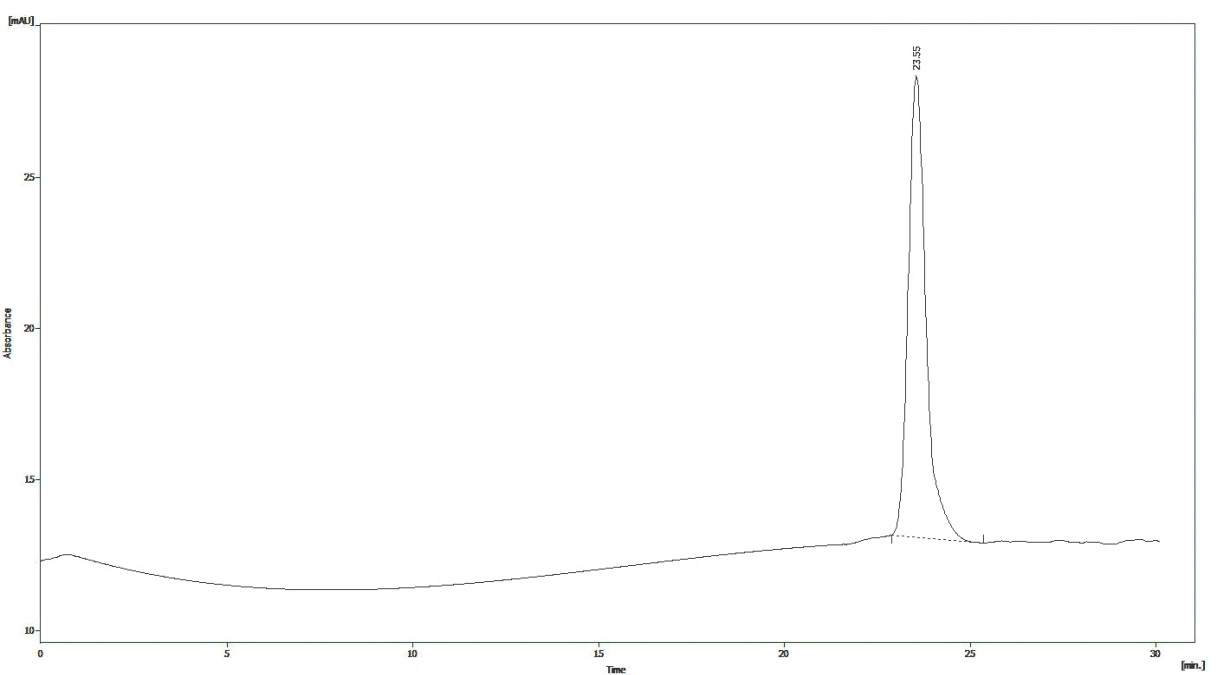


**Figure 2.** The HPLC chromatograms of diosgenin from the A) Fenugreek leaves and B) The standard with a concentration of 200 ppm, respectively.
