# Supplementary material for: Silymarin and serotonin, individually and synergistically, enhance fenugreek resistance to salt stress by modulating pathways involved in chlorophyll biosynthesis, defense system, hormonal regulation, ion redox balance, and diosgenin production
Source: BMC Plant Biol. 2026 Apr 17;26:929. doi: 10.1186/s12870-026-08765-7 (PMC13224543; doi:10.1186/s12870-026-08765-7)
Supplement: Supplementary file 2 — Supplementary Material 2. [file 12870_2026_8765_MOESM2_ESM.docx]

**Table S1**. Variance analysis of evaluated traits of plants under different levels of silymarin and serotonin

| SOV | df | Chl a | Chl b | ChlT | Car | RWC | MDA | ELI | Sol Pro | CAT |
| --- | --- | --- | --- | --- | --- | --- | --- | --- | --- | --- |
| Silymarin (Sm) | 2 | 63.37** | 16.63** | 144.93** | 20.07** | 616.85** | 30.27** | 325.92** | 17867.18** | 0.00513** |
| Serotonin (Ser) | 2 | 116.79** | 20.52** | 235.18** | 15.22** | 585.37** | 26.26** | 273.21** | 17806.51** | 0.00579** |
| Salinity (Sl) | 1 | 884.11** | 48.10** | 1344.68** | 42.86** | 3991.92** | 123.94** | 1854.80** | 600400.66** | 0.05181** |
| Sm*Ser | 4 | 1.45 ^ns^ | 0.04 ^ns^ | 1.08 ^ns^ | 0.06 ns | 5.21 ^ns^ | 0.11 ^ns^ | 6.91** | 711.76** | 0.00019** |
| Sm*Sl | 2 | 5.83** | 1.94** | 14.39** | 0.62** | 97.89** | 11.84** | 190.45** | 8124.66** | 0.00105** |
| Ser*Sl | 2 | 0.64 ^ns^ | 0.02 ^ns^ | 0.91^ns^ | 0.17* | 9.20** | 2.97** | 109.01** | 5318.00** | 0.0009595** |
| Sm*Se*Sl | 4 | 0.032 ^ns^ | 0.0089 ^ns^ | 0.06 ^ns^ | 0.02 ^ns^ | 14.03** | 0.16 ^ns^ | 8.00** | 908.58** | 0.00001 ^ns^ |
| Error | 36 | 0.625 | 0.02 | 0.61 | 0.03 | 1.04 | 0.10 | 1.11 | 32.88 | 0.00003 |

**Chla:** Chlorophyll a, **Chlb:** Chlorophyll b, **ChlT:** Total chlorophyll, **Car:** Carotenoid, **RWC:** Relative Water Content, **ELI:** Electrolyte Leakage Index, **MDA**: Malondialdehyde, **Sol Pro:** Total Soluble Protein**, CAT:** Catalase.

* and ** Significantly at the probability level of %5 and %1, respectively.

| **SOV** | **df** | **APX** | **GPX** | **SOD** | **PPO** | **Phe** | **Flav** | **K^+^** | **Na^+^** |
| --- | --- | --- | --- | --- | --- | --- | --- | --- | --- |
| **Silymarin (Sm)** | 2 | 0.00404** | 0.00366** | 0.00710** | 0.00651** | 2064.35** | 848.38** | 4.9086** | 4.729** |
| **Serotonin (Ser)** | 2 | 0.00631** | 0.00894** | 0.00781** | 0.00748** | 994.57** | 975.38** | 4.2828** | 4.418** |
| **Salinity (Sl)** | 1 | 0.05885** | 0.10374** | 0.07060** | 0.07343** | 11237.79** | 5995.57** | 13.5500** | 9.242** |
| **Sm*Ser** | 4 | 0.00003 ^ns^ | 0.0000057 ^ns^ | 0.00003** | 0.00014** | 62.26** | 19.61** | 0.1912** | 0.092** |
| **Sm*Sl** | 2 | 0.00161** | 0.00170** | 0.00154** | 0.00348** | 813.12** | 196.68** | 0.0654** | 0.598** |
| **Ser*Sl** | 2 | 0.00077** | 0.00179** | 0.00182** | 0.00132** | 279.01** | 137.79** | 0.0028 ^ns^ | 0.300** |
| **Sm*Se*Sl** | 4 | 0.00003 ^ns^ | 0.00003 ^ns^ | 0.00004** | 0.00006** | 43.43** | 17.07** | 0.0076^ns^ | 0.019** |
| **Error** | 36 | 0.00001 | 0.00001 | 0.0000063 | 0.00001 | 3.05 | 1.27 | 0.0055 | 0.004 |

**Table S1**. Variance analysis of evaluated traits of plants under different levels of silymarin and serotonin

**APX:** Ascorbate peroxidase, **SOD:** Superoxide dismutase, **GPX:** Guaiacol peroxidase, **APX:** Ascorbate peroxidase, **PPO:** Polyphenol oxidase, **Phe:** Phenol, **Flav:** Flavonoid, **K^+:^** Potassium, **Na^+^ :** Sodium

* and ** Significantly at the probability level of %5 and %1, respectively.

**Table S1**. Variance analysis of evaluated traits of plants under different levels of silymarin and serotonin

| **SOV** | **df** | **Sug** | **Aux** | **ABA** | **Prol** | **H_2_O_2_** | **NO** | ***SQS*** | ***SEP*** | ***CAS*** |
| --- | --- | --- | --- | --- | --- | --- | --- | --- | --- | --- |
| **Silymarin (Sm)** | 2 | 306.47** | 807.35** | 527.57** | 403.84** | 93.49** | 61.27** | 60.57** | 117.25** | 36.16** |
| **Serotonin (Ser)** | 2 | 145.97** | 642.57** | 177.57** | 198.34** | 98.66** | 56.08** | 96.04** | 79.67** | 59.18** |
| **Salinity (Sl)** | 1 | 489.00** | 1677.79** | 832.29** | 408.37** | 477.63** | 194.94** | 572.37** | 125.68** | 115.39** |
| **Sm*Ser** | 4 | 4.42** | 4.74* | 1.68 ^ns^ | 8.69** | 5.53** | 0.33 ^ns^ | 4.79** | 1.12* | 0.87* |
| **Sm*Sl** | 2 | 16.86** | 39.01** | 107.24** | 8.51** | 19.36** | 18.87** | 0.82 ^ns^ | 1.81* | 2.26** |
| **Ser*Sl** | 2 | 9.03** | 10.90** | 0.90 ^ns^ | 11.29** | 16.12** | 10.02** | 13.62** | 1.29 ^ns^ | 2.31** |
| **Sm*Se*Sl** | 4 | 0.72 ^ns^ | 2.46 ^ns^ | 1.18 ^ns^ | 1.13 ^ns^ | 5.79** | 0.31 ^ns^ | 1.39* | 0.10 ^ns^ | 0.81* |
| **Error** | 36 | 0.68 | 1.48 | 1.16 | 0.59 | 0.79 | 0.49 | 0.48 | 0.41 | 0.24 |

**Sug:** Sugar content, **Aux:** Auxin content, **ABA:** Abscisic acid content, **Prol:** Proline content, **H_2_O_2_:** Hydrogen peroxide, **NO:** Nitric oxide, ***SQS:*** Squalene synthase***, SEP:*** Squalene epoxidase***, CAS:*** Cycloartenol synthase

* and ** Significantly at the probability level of %5 and %1, respectively

**Table S1**. Variance analysis of evaluated traits of plants under different levels of silymarin and serotonin

| **SOV** | **df** | ***SSR*** | ***SMT*** | ***BGL*** | **Dios** |
| --- | --- | --- | --- | --- | --- |
| **Silymarin (Sm)** | 2 | 63.32** | 73.33** | 21.30** | 981.68** |
| **Serotonin (Ser)** | 2 | 81.84** | 62.23** | 33.94** | 1085.11** |
| **Salinity (Sl)** | 1 | 181.36** | 49.15** | 83.22** | 5735.04** |
| **Sm*Ser** | 4 | 1.12 ^ns^ | 0.09 ^ns^ | 0.08 ^ns^ | 24.54** |
| **Sm*Sl** | 2 | 0.09 ^ns^ | 0.21^ns^ | 1.07** | 49.31** |
| **Ser*Sl** | 2 | 1.20 ^ns^ | 0.89 ^ns^ | 0.91** | 46.44** |
| **Sm*Se*Sl** | 4 | 0.94 ^ns^ | 0.38 ^ns^ | 0.51* | 2.79 ^ns^ |
| **Error** | 36 | 0.44 | 0.40 | 0.14 | 2.02 |

***SSR:*** Squalene synthase reductase, ***SMT:*** Sterol methyltransferase, ***BGL:*** 26-o-Beta glucosidase, **Dios:** Diosgenin

* and ** Significantly at the probability level of %5 and %1, respectively

**Table S2.** Primers used in this study and their characteristics

| **Melting point (^°^C)** | **Length product (bp)** | **Reverse** | **Forward** | **Gene** |
| --- | --- | --- | --- | --- |
| 60 | 210 | CATTCTGTGTGTCTCCCTGCC | AGGTGGGAGATATGCTAGAATGGG | Squalene Synthase Reductase (*SSR*) |
| 60 | 230 | CACAGTAACTCATGACAACAGCAACCT | GAGAAGGCTGCAGAGGGTCTAG | Sterol Methyltransferase (*SMT*) |
| 61 | 220 | CAGATGCACCAGAGAGTAATCG | CTATTCTCAGAAGGACCGATCTC | Squalene epoxidase (*SEP*) |
| 60 | 230 | TACATGACGACGGTATTCTCCC | AAGAGAGATCCAACACCACTGC | Cycloartenol synthase (*CAS*) |
| 61 | 160 | TACCCACTGTTCCATTGCTATCC | GGCTCAACCATGATTCTCATACTG | Squalene synthase (*SQS)* |
| 60 | 210 | CACTGTATAACCTGCTGCCCA | GGCATTGATGAGTTCAACGATCC | 26-o-Beta glucosidase (*BGL*) |
| 61 | 230 | ATGTTAAATGATGCAGCCCTTCCACCTCTC | TATGTTTGTTGTTGGTGTCAACGAGCAACGAATACAAG | Glyceraldehyde 3-phosphate dehydrogenase-Reference gene *(GAPDH)* |
